# Efficacy and safety of antireflux surgery in gastroesophageal-related cough: a systematic review and meta-analysis

**DOI:** 10.1097/JS9.0000000000001998

**Published:** 2024-08-30

**Authors:** Yuheng Liu, Junfeng Huang, Shuxin Zhong, Ziwen Zheng, Zhixing Xu, Dongda Zhou, Shuojia Xie, Zikai Lin, Shiyue Li, Ruchong Chen

**Affiliations:** a Guangzhou Institute of Respiratory Health, State Key Laboratory of Respiratory Disease, National Clinical Research Center for Respiratory Disease, National Center for Respiratory Medicine; b Department of Allergy and Clinical Immunology, The First Affiliated Hospital of Guangzhou Medical University; c Clinical Medical College, Guangzhou Medical University, Guangzhou, Guangdong, People’s Republic of China

**Keywords:** antireflux surgery, cough, gastroesophageal reflux disease (GERD), reflux symptoms, systematic review

## Abstract

**Background:**

Gastroesophageal reflux disease (GERD) is a prevalent condition that manifests a spectrum of symptoms, including gastroesophageal-related cough (GERC). Antireflux procedures have been employed to alleviate these symptoms, yet their efficacy varies. This systematic review and meta-analysis aim to evaluate the improvement in gastroesophageal-related cough and other reflux symptoms following antireflux procedures.

**Methods:**

A systematic review was performed by searching PubMed, Embase, and Cochrane Library. All observational studies reporting the improvement of GERC and other reflux symptoms after the antireflux procedures. Data were extracted and pooled using a random effects model to assess the overall effect size and heterogeneity between studies. The authors found that antireflux surgery has some clear benefits for common reflux-related symptoms.

**Results:**

Fifty-nine eligible studies with 7431 patients with GERD were included in this study. The pooled cough remission rate was 80.0% (95% CI: 75.4–84.2) and the mean time of follow-up was 35.8 months. Antireflux surgery significantly improved overall reflux-related symptom scores (all *P*<0.001). The authors also assessed the rate of remission of other reflux symptoms. The pooled heartburn remission rate was 87.7% (95% CI: 82.3–92.2) and the pooled regurgitation remission rate was 91.2% (95% CI: 87.8–94.1).

**Conclusion:**

Antireflux procedures significantly improve gastroesophageal-related cough and other reflux symptoms. These findings support the use of antireflux procedures as a viable treatment option for patients with GERD symptoms. Further research is needed to identify predictors of success and to optimize patient selection for antireflux procedures.

## Introduction

Highlights

- PPIs and antireflux procedures have been employed in GERD.
- Antireflux procedures significantly improve gastroesophageal-related cough and other reflux symptoms.
- Antireflux surgery significantly improved total reflux-related symptom scores.
- Nissen fundoplication had less perioperative symptoms than patients who had other types of surgery.
- Antireflux surgery was a relatively safe treatment for GERD.

Gastroesophageal reflux disease (GERD) was characterized by the regurgitation of stomach acid and food into the esophagus, and affected ~13.98% of the global population worldwide^1,2^. GERD is a potential cause of chronic cough (up to 8 weeks duration). Besides, gastroesophageal-related cough (GERC) is one of the most common types of chronic cough. Twenty percent of patients with chronic cough were GERC^3^. Patients with GERC tend to have a long disease course and a low quality of life. Currently, lifestyle modification and antireflux therapy with medications are generally identified as first-line treatment for GERC in different guidelines and reviews^4–7^. However, some patients with GERC may not respond effectively or are intolerant to long-term antireflux medications. To improve this situation, both GERC and GERD therapy guidelines recommend surgical or endoscopic antireflux procedures as an effective treatment for GERC in patients with objective reflux evidence^4,6,7^.

Antireflux procedure involves wrapping the upper part of the stomach around the lower end of the esophagus to create a new valve-like mechanism. The most common type of antireflux surgery is laparoscopic Nissen fundoplication, which has been shown to be effective and safe in improving symptoms and quality of life in patients with GERD^8^. Other types of antireflux surgery include partial fundoplication, Toupet fundoplication, or LINX device implantation. Antireflux procedure offers an alternative to medical therapy that may minimize chronic antireflux medications [like proton pump inhibitors (PPIs)] use and its associated risks. Previous systematic review indicated that antireflux surgery offers superior short-term symptom control and higher quality of life than medical treatment of GERD^9^.

Although antireflux surgery has been widely used for controlling GERD’s symptoms, the lack of research on the patients with GERC makes the efficacy and safety for treating GERC remain controversial. The aim of this study is to provide the current evidence on the efficacy and safety of antireflux surgery for patients with GERC and to inform the therapy of the individuals suffering GERC.

## Methods

### Search strategy

A systematic literature search of electronic databases, including Medline, Embase, Cochrane Library, and ClinicalTrials.gov was performed from their inception until March 2023. The search strategy used the following terms: ((‘GERD’) OR(‘GORD’) OR (‘gastroesophageal reflux disease’)) AND ((‘anti-reflux surgery’) OR (‘surgery’)). In addition, we conducted this study following the guidelines of the Preferred Reporting Items for Systematic Reviews and Meta-Analyses (PRISMA, Supplemental Digital Content 1, http://links.lww.com/JS9/D379, Supplemental Digital Content 2, http://links.lww.com/JS9/D380) statement^10^ and assessing the methodological quality of systematic reviews (AMSTAR) (Supplemental Digital Content 3, http://links.lww.com/JS9/D381) Guidelines^11^.

### Selection criteria

Observational studies providing relevant data for patients with GERD after the antireflux surgery were included. The titles and abstracts were reviewed for relevance to the topic, and the full text were assessed to determine eligibility. Eligible articles were identified if they met the following inclusion criteria: (1) Studies that reported incidence and improvement of cough in the patients with GERD who underwent the antireflux surgery; (2) Studies that reported incidence and improvement of other reflux symptoms in the patients with GERD who underwent the antireflux surgery. We excluded studies that were not published as full reports, such as conference abstracts and letters to editors, and that did not report the outcomes of interest.

### Data extraction

Each included article was independently reviewed by two researchers, and a third researcher would confirm the data extraction in case of any discrepancies. Extracted data included study characteristics (first author, country, sample size, and so on), patients’ demographics, incidence, and improvement of reflux symptoms before and after the surgery.

### Quality and risk of bias assessment

The Newcastle–Ottawa Scale (NOS) was used to assess the methodological quality of observational studies^12^. The NOS scores of 0–3, 4–6, and 7–9 were assigned for low, moderate, and high-quality studies, respectively. We used the Grading of Recommendations Assessment, Development and Evaluation (GRADE) system to appraise the quality of the evidence for incidence, mortality, and each risk factor^13^. The certainty of evidence was categorized as high, moderate, low, and very low.

### Statistical analysis

A random-effects model was used for all the outcomes. Weighted mean differences and standard mean differences were used for the analysis of continuous. The pooled incidence and remission would further be classified into multiple subgroups. Heterogeneity among studies was measured using Cochran’s *Q* and *I* ^2^ statistics, and significant heterogeneity was defined as *I* ^2^>50% and *P-*value <0.1 in the Q-test. The publication bias was detected with funnel plots, Egger’s, and Begg’s test for pooled results with more than 10 included studies. Additionally, subgroup analysis and meta-regression were performed to explore potential sources of heterogeneity. All statistical analyses were conducted with STATA (version 15.1; Stata Corporation).

## Results

### Study characteristic and quality assessment

**Figure 1:**
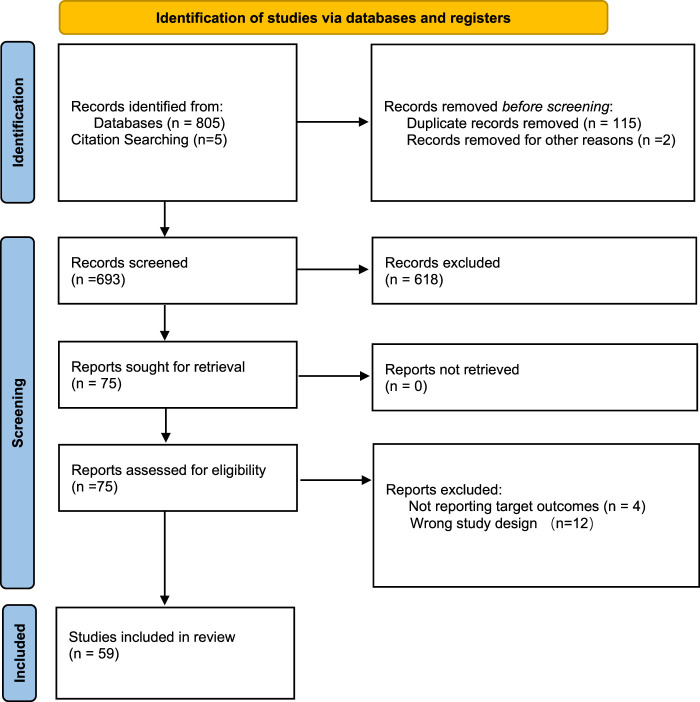
Flowchart for study inclusion and exclusion.

A comprehensive search of the online databases initially retrieved 810 potentially relevant studies. After carefully screening the titles/abstracts and full texts, 752 articles were excluded. Finally, 59 studies, with a total of 7431 patients, met the criteria and were included in the meta-analysis. The PRISMA flowchart of literature search and selection is presented in Figure 1. A majority (7032, 95%) of patients with GERD underwent antireflux surgery, of whom 3577 (48%) had a chronic cough before surgery. All eligible studies reported a cough improvement rate and/or a change in cough symptom scores before and after surgical treatment. The study population was also adequately described in all studies. The mean age of the included patients was 50.40±2.59 years, and the mean cough duration was 6.21±2.78 months. The laparoscopy approach was performed in 80% of the studies (50/60), with the remaining surgical approaches being open or unknown. The most common type of surgery was the Nissen fundoplication, followed by other procedures (Toupet, Stretta, etc.) and combined surgery. In addition, 15 studies reported the use of perioperative antireflux treatments such as PPIs and *H2*-receptor antagonists. The baseline characteristics of all trials and included patients are shown in Table S1 (Supplemental Digital Content 4, http://links.lww.com/JS9/D382), respectively. Moreover, random-effects models were used for all overall and subgroup analyses in this study.

The present systematic review included RCTs, cohorts, and case–control studies. The 59 included studies were primarily single-center and had a median sample size of 91 (range 8–950). The overall quality of the 58 observational studies was high, with a Newcastle–Ottawa score range of 4–9 (Table 1). Additionally, all intervention studies (two RCTs and three trials) met the high-quality criteria of the Cochrane tool. The results of the quality evaluation for the prospective trials are presented as a risk of bias graph and a summary risk of bias figure in Figure S1 (Supplemental Digital Content 4, http://links.lww.com/JS9/D382).

**Table 1:** Characteristics of the studies included in the meta-analysis.

n.	Study	Year	Area	No. of patient	Design	Center	Cough improvement rate	Score
1	Frankel	2022	Singapore	66	Cohort	Multi	61%	9
2	Gisi	2021	USA	33	Cohort	Single	60%	6
3	Aiolfi	2020	USA	86	Cohort	Single	88%	7
4	Dong	2019	China	79	Case–control	Multi	60%	6
5	Park	2019	USA	232	Cohort	Single	76%	8
6	Esposito	2018	Australia	220	Cohort	Single	92%	7
7	Kalapala	2017	India	20	RCT	Single	/	–
8	Andolfi	2016	USA	165	Cohort	Single	94%	8
9	Liang	2015	China	165	Cohort	Single	/	8
10	Yan	2015	China	98	Cohort	Multi	71%	7
11	Adaba	2014	UK	208	Case–control	Single	78%	7
12	Drews	2014	Germany	85	Cohort	Single	93%	6
13	Liang	2014	China	152	Cohort	Single	/	7
14	Lugaresi	2014	Italy	150	Cohort	Single	85%	7
15	Toomey	2014	USA	60	Case–control	Single	/	6
16	Liang WT	2014	China	122	Cohort	Multi	/	8
17	Patti	2013	USA	49	Cohort	Single	33%	4
18	Kiljander	2012	Finland	69	Clinical trial	Multi	/	–
19	Hoppo	2013	USA	16	Cohort	single	100	8
20	Koch	2012	Germany	100	RCT	Single	78%	–
21	Zhang	2012	China	198	Cohort	Multi	87%	8
22	Brown	2011	USA	113	Cohort	Single	50%	8
23	Trad	2011	USA	28	Cohort	Multi	79%	5
24	Wassenaar	2011	USA	10	Cohort	Multi	50%	6
25	Westhuizen	2011	USA	244	Cohort	Single	76%	7
26	Bott	2010	UK	21	Cohort	Single	95%	5
27	Iqbal	2008	UK	40	Cohort	Single	84%	5
28	Oelschlager	2008	USA	288	Cohort	Multi	69%	9
29	White	2008	USA	22	Clinical trial	Single	38%	–
30	Ranson	2007	USA	91	Cohort	Single	76%	7
31	McClusky	2007	USA	8	Cohort	Single	/	5
32	Kaufman	2006	USA	128	Cohort	Single	74%	7
33	Liu	2006	USA	43	Cohort	Single	89%	5
34	Swoger	2006	USA	72	Cohort	Multi	67%	7
35	Ciovica	2005	Australia	126	Cohort	Multi	97%	8
36	Ziora	2005	Poland	65	Case–control	Multi	85%	6
37	Allen	2004	Canada	905	Cohort	Single	71%	9
38	Brouwer	2003	Australia	102	Cohort	Single	95%	8
39	Wright	2003	USA	145	Case–control	Multi	92%	8
40	Hui	2002	USA	100	Cohort	Single	60%	8
41	Allen	2002	Canada	677	Case–control	Single	85%	6
42	Greason	2002	USA	65	Clinical trial	Single	60%	–
43	Irwin	2002	USA	8	Cohort	Single	100%	5
44	Lindstrom	2002	USA	29	Cohort	Single	88%	5
45	Novitsky	2002	USA	21	Cohort	Single	86%	5
46	Oelschlager	2002	USA	21	Case–control	Single	100%	7
47	Thoman	2002	USA	129	Cohort	Single	65%	6
48	Farrell	2001	USA	324	Cohort	Single	96%	6
49	Ackroyd	2001	Australia	82	Cohort	Single	61%	5
50	Ekström	2000	Sweden	24	Cohort	Single	100%	5
51	Patti	2000	USA	39	Cohort	Multi	74%	6
52	Tibbling	1993	Sweden	208	Cohort	Single	81%	7
53	Allen	1998	USA	195	Cohort	Single	83%	5
54	Wetscher	1997	Australia	21	Cohort	Single	100%	6
55	Anvari	1996	Canada	69	Cohort	Single	73%	7
56	Hunter	1996	Georgia	300	Cohort	Single	81%	5
57	Johnson	1996	USA	50	Cohort	Single	76%	7
58	Ribet	1989	France	132	Cohort	Single	68%	6
59	Lomasney	1977	USA	129	Cohort	Single	100%	6

* RCT, randomized controlled trial.

# The quality of these studies was assessed by using the Cochrane Collaboration Tool.

### Improvement and incidence in cough symptoms after antireflux surgery

Forty-five studies, including a total of 3013 patients reported improvement in cough after antireflux surgery, and the pooled incidence of cough symptoms was 58.2% (95% CI: 49.4–66.7). As shown in Table 2, the pooled cough improvement rate (CIR) was 80.0% (95% CI: 75.4–84.2) during a mean follow-up of 35.8 months. Both the Cochran *Q* test and the *I* ^2^ statistic revealed significant heterogeneity (*I* ^2^ *=*86.09%, *P*<0.001), with CIRs ranging from 33 to 100% in each study.

**Table 2:** Cough remission rate.

Variable	Study (*n*)	No. of patients	Patients with cough remission	Pooled remission rate (95% CI)	Certainty in the pooled estimate
Total	45	3013	2341	80.0% (75.4–84.2)	Low
Area	44	2965	2297		
North America	31	2091	1592	78.9% (72.9–84.5)	Low
Europe	9	474	399	85.5% (77.0–92.3)	Low
Asia	4	400	306	73.2% (56.2–87.4)	Very low
Procedure	33	2605	1983		
Nissen	26	2321	1798	79.2% (73.8–84.2)	Low
Toupet	2	97	85	87.6%	Very low
Other procedure	5	187	100	83.0% (67.1–94.7)	Very low
Type	37	2708	2066		
Laparascope	35	2681	2046	80.8% (76.2–85.0)	Very low
Other	2	27	20	74.1%	Very low
Follow-up	27	2757	2241		
<6 months	11	1272	1092	86.1% (81.1–90.4)	Low
6–12 months	9	570	486	87.0% (78.4–93.9)	Low
>12 months	7	915	663	72.4% (67.3–77.3)	Low
Perioperative treatment	47	3313	2495		
Yes	23	1367	1062	83.1% (75.7–89.5)	Low
No	24	1946	1433	76.3% (69.1–83.0)	Low

* Other in type, including undefined type and studies with a small size sample; TIF, transoral incisionless fundoplication.

Subgroup analyses were performed based on area, type of surgery, duration of follow-up, and perioperative medication use (Figures S2–S5, S10, Supplemental Digital Content 4, http://links.lww.com/JS9/D382). First, to assess the long-term outcome after antireflux surgery, studies with different follow-up times were divided into three groups: short-term (<6 months), mid-term (6–12 months), and long-term (>12 months). The pooled CIR in studies with medium-term follow-up (87.0%, 95% CI: 78.4–93.9) was significantly higher than in those with long-term follow-up (72.4%, 95% CI: 67.3–77.3; *P<*0.001). Additionally, patients who received perioperative antireflux treatment (83.1%, 95% CI: 75.7–89.5) had a higher CIR than those who did not (76.3%, 95% CI: 69.1–83.0; *P<*0.001). These results indicate that long follow-up periods and nonuse of perioperative treatment might be associated with worse CIR, although substantial heterogeneity was observed (Both *I* ^2^>50%). On the contrary, the CIR showed no significant difference in terms of continents and surgical procedures, as the 95% CI overlapped extensively.

**Figure 2:**
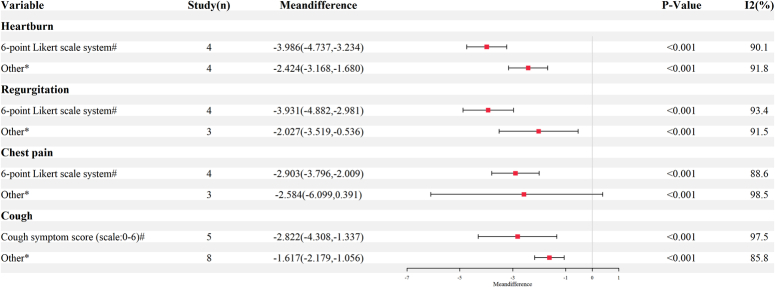
Forest plots of assessment of symptom score change after antireflux surgery (#, weighted mean difference; *, standard mean difference).

Fifty-six studies provided a quantitative assessment of cough symptoms via subjective questionnaires, such as visual analog scale (VAS) score, cough symptom score (CSS), Leicester Cough Questionnaire (LCQ), etc. (Figure S11, Supplemental Digital Content 4, http://links.lww.com/JS9/D382). As shown in Figure 2, the pooled results showed that antireflux surgery was associated with a significant reduction of cough symptom score (range 0–6) for cough symptoms (WMD=−2.82, 95% CI: −4.31 to −1.34, *P*<0.001). Meanwhile, other types of scales (CCS, LCQ, etc.) were grouped due to the insufficient number of studies and different score ranges. Similarly, other scales of cough symptoms were also significantly decreased after antireflux surgery (SMD= −1.62, 95% CI: −2.18 to −1.06, *P*<0.001), but significant heterogeneity was observed in both analyses (Both *I* ^2^>50%).

### Improvement and incidence in reflux symptoms after antireflux surgery

A total of 53 studies encompassing 3078 patients assessed improvement of multiple reflux symptoms. The detailed results on incidence and improvement of reflux symptoms were shown in Table 3, Table 4, and Figures S6–S9 (Supplemental Digital Content 4, http://links.lww.com/JS9/D382). In terms of incidence of symptoms, the most common symptoms involved heartburn (82.9%) and regurgitation (72.0%). Among other respiratory extra-esophageal symptoms, the incidence of wheeze was 36.8% (95% CI: 11.9–66.2), and the incidence of hoarseness was 43.3% (95% CI: 20.1–68.2). These results suggested that cough may be the most common extra-esophageal symptom, only less than heartburn [82.9%, 95% CI (74.4–90.0)] and regurgitation [72.0%, 95% CI (54.6–86.6)]. As to remission of symptoms, the most commonly reported symptoms of reflux included heartburn (87.7%), followed by regurgitation (77.9%), chest pain (39.4%), dysphagia (34.2%), and nausea (24.7%). As expected, the overall pooled improvement rate for each of the above reflux symptoms (except for wheeze and bloating) after antireflux surgery was more than 65%. To be precise, the postoperative improvement rates of heartburn and regurgitation symptoms were 87.7% (95% CI: 82.3–92.2) and 91.2% (95% CI: 87.9–94.1), respectively. For the symptom of chest pain, 72.1% (95% CI: 53.2–88.2) of patients reported improvement after antireflux surgery. Also, the remission rate of dysphagia and nausea symptoms was relatively significant after the procedure. Among other respiratory GERD-related symptoms, 67.4% (95% CI: 60.5–73.9) of patients with bloating were relieved after antireflux surgery. As with cough symptoms, other common reflux symptom scores were assessed in this study. As shown in Figure 2, antireflux surgery significantly improved total reflux-related symptom scores in total. Furthermore, the 6LS scale scores for reflux, heartburn, and chest pain all improved significantly postoperatively (all *P*<0.001). As expected, antireflux surgery has some definite efficacy for the common reflux-related symptoms mentioned above.

**Table 3:** Remission rate of GERD complications.

					Heterogeneity	
Variable	Study (*n*)	No.of patients	Pooled remission rate	95% CI	*I* ^2^ (%)	*P*
Heartburn	16	1250	87.7	(82.3–92.2)	83.42	<0.001
Epigastric pain	3	206	100.0	(97.9–100.0)	0.00	0.485
Regurgitation	15	974	91.2	(87.8–94.1)	57.66	0.003
Dysphagia	9	405	73.6	(63.7–82.5)	70.29	0.001
Chest pain	6	144	72.1	(53.2–88.2)	71.33	0.004
Wheeze	2	54	35.0	/	/	/
Hoarseness	4	205	67.4	(60.5–73.9)	0.00	0.64
Bloating	1	54	46.0	/	/	/
Nausea	4	99	74.3	(61.0–86.0)	28.57	0.241

**Table 4:** Incidence rate of GERD complications.

					Heterogeneity	
Variable	Study (*n*)	No.of patients	Pooled incidence rate	95% CI	*I* ^2^ (%)	*P*
Cough	58	7496	58.2	(49.4–66.7)	98.2	<0.001
Heartburn	16	1614	82.9	(74.4–90.0)	93.7	<0.001
Epigastric pain	3	292	57.0	(16.4–92.8)	97.9	<0.001
Regurgitation	15	1656	72.0	(54.6–86.6)	98.1	<0.001
Dysphagia	10	1069	57.4	(36.6–77.0)	97.8	<0.001
Chest pain	8	810	34.5	(22.4–47.6)	92.0	<0.001
Wheeze	4	620	36.8	(11.9–66.2)	98.0	<0.001
Hoarseness	6	842	43.3	(20.1–68.2)	97.8	<0.001
Diarrhea	1	69	58.0	/	/	/
Bloating	1	69	78.3	/	/	/
Nausea	5	436	28.5	(17.5–40.9)	85.4	<0.001

### Analysis of surgical complications

**Figure 3:**
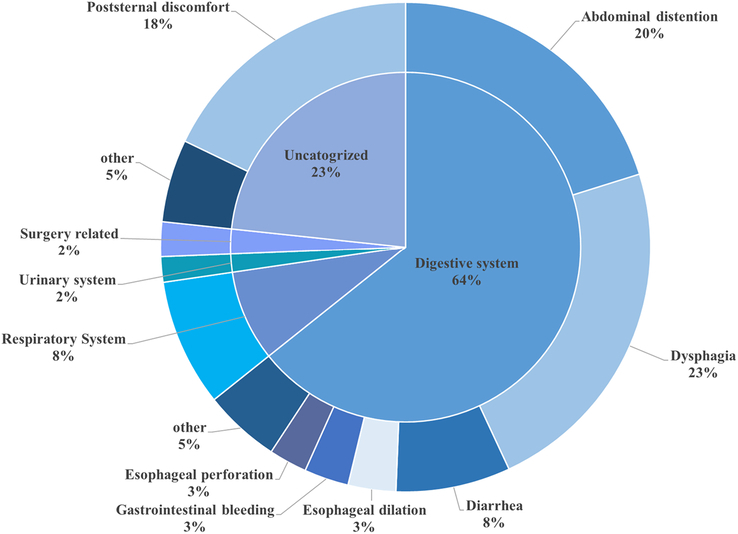
Pie chart showing the distribution of the perioperative complications.

A total of 23 studies, including 3291 patients reported on data on surgical complications, the details are shown in Table 5. In those studies, the patients mainly underwent laparoscopic Nissen fundoplication. 14.1% (6.0–24.7) of them had perioperative symptoms (*n*=464). As shown in Figure 3, the digestive system symptoms accounted for the most in total (64%). Among them, dysphagia occupied 23% and abdominal distention occupied 20% in total perioperative symptoms. Besides these, poststernal discomfort is the third most symptoms in all perioperative complications (18%). Compared with other type of surgery, patients who had laparoscopic Nissen fundoplication had less perioperative symptoms than patients who had other types of surgery (6.6% vs 7.6%. *P*<0.05).

**Table 5:** Perioperative complications.

Variable	Number (%)
Rate	14.1 (6.0–24.7)
Total	464 (100%)
Type (95% CI)	
Nissen	6.6% (2.3–12.6)
Other	7.6% (4.0–12.1)
Digestive system	
Abdominal distention	96 (20.69%)
Diarrhea	36 (7.76%)
Abdominal pain	1 (0.22%)
Oral intolerance	4 (0.86%)
Esophageal hiatus	1 (0.22%)
Esophageal perforation	12 (2.59%)
Esophageal dilation	15 (3.23%)
Transhiatal hernia	3 (0.65%)
Intestinal obstruction	3 (0.65%)
Gastric perforation	6 (1.29%)
Folding hernia of gastric fundus	1 (0.22%)
Dysphagia	109 (23.49%)
Gastrointestinal bleeding	14 (3.02%)
Peritonitis	1 (0.22%)
Splenogastric short vessel hemorrhage	1 (0.22%)
Slight liver laceration	3 (0.65%)
Respiratory system	
Sore throat	9 (1.94%)
Respiratory acidosis	3 (0.65%)
Chylothorax	1 (0.22%)
Pneumonia	6 (1.29%)
Pneumothorax	5 (1.08%)
Pulmonary embolism	3 (0.65%)
Bronchiectasia	1 (0.22%)
Urinary system	
Urinary retention	6 (1.29%)
Urinary tract infection	2 (0.43%)
Surgery-related	
ELGP related	3 (0.65%)
Nissen slip	4 (0.86%)
Tight wrap requiring endoscopic expansion	4 (0.86%)
Other	
Intracranial hemorrhage	1 (0.22%)
Hoarseness	1 (0.22%)
Laryngitis	1 (0.22%)
Arrhythmia	8 (1.72%)
Wound infection	9 (1.94%)
Poststernal discomfort	85 (18.32%)
Fever	6 (1.29%)

### Meta-regression: a multivariate analysis

In order to investigate potential factors affecting the heterogeneity of cough remission rate and reflux symptom remission rate, multivariate meta-regression technique was used (Table S2, Supplemental Digital Content 4, http://links.lww.com/JS9/D382). There was no significant relationship between cough remission rate and year, the number of patients or sex ratio even in subgroup. Similarly, year, the number of patients, or sex ratio also could not affect reflux symptom remission rate even in subgroup.

### Publication bias and sensitivity analysis

We conducted publication bias and sensitivity analysis on cough remission rate, reflux symptom remission rate, and symptom score change (Figures S12–S17, Supplemental Digital Content 4, http://links.lww.com/JS9/D382). According to Begg’s test and Egger’s test, publication bias in most of them was not significant. Besides, all sensitivity analyses produced null results.

## Discussion

Chronic cough is the most difficult and controversial symptom associated with GERD, and has a relatively high incidence compared to other extraesophageal symptoms^14–16^. However, there have not been enough evidence to provide guidance on clinical treatment^17^. This systematic review and meta-analysis comprehensively analyzed the pooled incidence of cough in patients with antireflux surgery, and confirmed the remission indication for cough and surgery-related complications. The present study found that antireflux procedure demonstrated a beneficial effect on relieving cough symptoms, especially in those undergoing acid-suppression therapy, and this effect diminished over time. In terms of safety, this research pooled the reported incidence of each type of complications after surgery. To our knowledge, this is the first meta-analysis to evaluate both the efficacy and safety of antisurgery for GERC. Overall, our study may provide evidence for the treatment strategy in patients with refractory GERC.

Our study showed that the overall incidence of cough is 58.2% (95% CI: 49.4–66.7) in patients with GERD and 48.0% in patients underwent surgery, consistent with the relatively high rates in previous studies^9,14,18,19^. In the present study, the incidence of cough was the third highest after the typical symptom for GERD (heartburn and regurgitation). Our results indicated cough symptoms appear to be the most common respiratory complication of GERD, which was also mentioned in other studies^20,21^. In addition to high incidence, cough is also regarded as hard to relieve in clinical scenarios^17^. Other less common symptoms mentioned in published studies were hard to conduct analysis due to the lack of data^16^. Moreover, our results demonstrated that the remission rate of cough decreased with postoperative time, which were consisting with previous studies^22,23^. It may indicate that the follow-up time of the study may impact the reflux symptoms remission rate. Currently, the long-term outcome of reflux-related respiratory complications after laparoscopic antireflux surgery remains controversial. Several studies indicated that it may be associated with surgical types, perioperative antireflux medication, and various evaluations of symptoms^24,25^. Notably, the comparison between placebo or PPI and antireflux surgery in the improvement of chronic cough is worth further investigating^19^. Hence, a more comprehensive relationship between postoperative time and the remission rate or reoccurrence of manifestations in GERD needs to be explored in future studies. We also found that other reflux systems were also relieved to some extent. Several articles indicated that GERD patients with the manifestation of regurgitation and heartburn possessed better efficiency of antireflux surgery^26–28^. However, this result was not confirmed in our study due to the lack of relevant studies included. In the present study, it seems that cough in respiratory GERD complications has the highest remission rate. Furthermore, Tustumi *et al*.’s^29^ study has indicated that there was no significant difference in remission rate among cough, hoarseness, wheezing, and oral corticosteroid reduction after antireflux surgery. Prospective trials could be conducted in the future to compare the remission rate of GERC and other respiratory symptoms. We can also find the cough remission rate was higher than the remission rate of other extraesophageal symptoms, indicating that antireflux surgery was able to relieve symptoms effectively, especially for cough. Overall, this was an effective and reliable surgical technique for GERD but the long-term effectiveness was unknown. More studies with long-term follow-up were still needed. In addition, our results indicated large inter-individual differences, and therefore, more emphasis should be put on the characteristics of the beneficiary population.

In terms of the surgery type, almost all types of surgery techniques were laparoscopic. These results are similar to those reported by others. According to some studies, the most commonly used procedure was Nissen^4,30^, which was consistent with our findings. Furthermore, we indicated that the GERD patients treated with Toupet surgery had the highest remission rate of cough, while there were only two studies included. Thus, we needed more studies to demonstrate which procedure was more efficient and safer. Besides the procedure mentioned in our study, modified Nissen procedures are now worth continuing to explore in the future because of its potential to reduce the failure of traditional Nissen surgery and to treat the morbid obesity after the antireflux surgery^31–33^.

Our findings specifically indicated that cough remission rate in the subgroup with the perioperative treatment was higher than that without the perioperative treatment, though we were lack of the data of the direct comparison. The common perioperative treatment of antireflux surgery was PPI treatment, and many studies also utilized antireflux diet for weight management^4,34–36^. Our findings provided sufficient evidence to conduct perioperative treatment for those GERD patients who had surgery.

In previous studies, there was no significant difference between placebo and PPI in the resolution of GERC^37^. A systematic review has also demonstrated that PPIs slightly decrease the severity of nonspecific cough but did not achieve a clinically significant difference^38^. These results may suggest that antireflux surgery appears to be more appropriate for refractory GERC than medication alone. It remains to be investigated whether or how to use PPI for GERC as a primary treatment strategy. On the other hand, apart from medication, conservation therapy for GERD also involves lifestyle changes and breathing exercises. In previous studies, lifestyle change was regarded as an important part of the treatment of patients with reflux symptoms^39,40^. In addition, quality of life improved significantly in the breathing exercise group (13.4±1.98 before and 10.8±1.86 after breathing exercise), which may be a potential treatment for PPI-refractory GERD patients^41^. Speech pathology also served as a potential option for GERC, in terms of its efficacy in chronic cough^42^. In a multicenter randomized control trial, cough frequency decreased by 41% (95% CI: 36–95%) in the speech therapy group compared to control^43^. However, only a few researches of conservation therapy were conducted for remission of GERC. It is well worth pursuing in future studies to compare conservative and surgical treatments.

Antireflux surgery has been proven to be superior to medical treatment in highly selected patients^44,45^, especially for those with refractory GERD^46^. However, there was only a few evidence on which populations are suitable for receiving antireflux surgery for GERC^47^. Moreover, the lack of high-quality studies limits our further exploration of the population of benefit. Consequently, it is necessary to conduct more randomized clinical trials to further investigate the effectiveness of antireflux surgery compared to different medications. In addition, the optimal strategy for antireflux surgery treating GERC is also needed to be further explored in the future^9^. Types of antireflux surgery, perioperative management, and appropriate evaluation of cough symptoms remain need to be investigated in prospective studies.

However, there were several limitations in our study. First, we lack the study about the recurrence of the symptoms, thus we cannot precisely estimate the long-term effect of surgery. Second, there was no comparison of the effect between different types of surgery. Finally, various measurements with significant heterogeneity were used to assess the severity of cough in our included studies. Although the standard mean differences model was used employed to pool these data, this may inevitably lead to bias in the results. Hence, we could develop and validate a specific questionnaire for GERC evaluation in order to properly assess the efficacy of GERC after antireflux surgery. Despite these limitations, this study was conducted in strict accordance with the PRISMA and ISHIS consensus and represents the best currently available evidence.

‘Our meta-analysis suggested that antireflux surgery could effectively reduce the symptoms of the cough and other reflux symptoms. Our study also provided evidence to support a safe and effective surgical treatment for patients with GERC.’

## Ethical approval

Not applicable.

## Consent

Not applicable.

## Source of funding

This study is sponsored by the National Natural Science Foundation of China (82170024), Guangzhou Basic Research Program (2024A03J1142).

## Author contribution

Y.L., J.H., S.Z., and Z.Z.: study design; Y.L., J.H., and Z.Z.: literature search; S.Z., Z.X., D.Z., S.X., and Z.L.: data extraction; Y.L., J.H., Z.Z., and Z.L.: data analysis and quality assessment; Y.L., J.H., and S.Z.: manuscript writing; R.C. and S.L.: project guidance and funding acquisition. All authors read and approved the final manuscript and contributed in results interpretations.

## Conflicts of interest disclosure

All authors state that no financial and personal relationships with other people or organizations that could inappropriately influence (bias) their work.

## Research registration unique identifying number (UIN)

1. Name of the registry: PROSPERO.
2. Unique identifying number or registration ID: CRD42021287805.
3. Hyperlink to your specific registration (must be publicly accessible and will be checked): crd.york.ac.uk/PROSPERO/display_record.php?RecordID=287805.

## Guarantor

Ruchong Chen.

## Data availability statement

The authors confirm that the data supporting the findings of this study are available within the article [and/or its supplementary materials].

## Provenance and peer review

Not commissioned, externally peer-reviewed.

## Assistance with the study

Not applicable.

## Supplementary Material
