# Supplementary material for: Efficacy and safety of antireflux surgery in gastroesophageal-related cough: a systematic review and meta-analysis
Source: Int J Surg. 2024 Aug 30;111(1):1348–56. doi: 10.1097/JS9.0000000000001998 (PMC11745681; doi:10.1097/JS9.0000000000001998)
Supplement: Supplementary file 2 [file js9-111-1348-s002.docx]

**Figure 1** Flow chart for study inclusion and exclusion
