# Supplementary material for: Efficacy and safety of antireflux surgery in gastroesophageal-related cough: a systematic review and meta-analysis
Source: Int J Surg. 2024 Aug 30;111(1):1348–56. doi: 10.1097/JS9.0000000000001998 (PMC11745681; doi:10.1097/JS9.0000000000001998)
Supplement: Supplementary file 4 [file js9-111-1348-s004.docx]

**Supplement**

**(Forest plots of each analysis for surgical complications and risk factors，Publication bias of each analysis for surgical complications and risk factors)**

**The list of included studies**

**e-Figure 1** Risk of bias assessment

**e-Figure 2** Forest plots of assessment of cough improvement after anti-reflux surgery.

**e-Figure 3** Forest plots of assessment of cough improvement in different follow-up time after anti-reflux surgery.

**e-Figure 4** Forest plots of assessment of cough improvement in North America after anti-reflux surgery.

**e-Figure 5** Forest plots of assessment of cough improvement in different areas after anti-reflux surgery.

**e-Figure 6** Forest plots of the assessment of chest pain improvement after anti-reflux surgery.

**e-Figure 7** Forest plots of the assessment of heartburn improvement after anti-reflux surgery.

**e-Figure 8** Forest plots of the assessment of regurgitation improvement after anti-reflux surgery.

**e-Figure 9** Forest plots of assessment of different symptoms improvement after anti-reflux surgery.

**e-Figure 10** Forest plots of the association between the surgical techniques of anti-reflux surgery and the improvement of cough score in patients. (A)Nissen. (B) Other techniques.

**e-Figure 11** Forest plots of the improvement of cough score in patients who underwent laparoscopic surgery

**e-Figure 12** Funnel plot of log odd ratio (horizontal axis) and the standard error for the log odd ratio (vertical axis) for assessment of cough improvement after anti-reflux surgery.

**e-Figure 13** Funnel plot of log odd ratio (horizontal axis) and the standard error for the log odd ratio (vertical axis) for assessment of cough improvement in different follow-up time after anti-reflux surgery.

**e-Figure 14** Funnel plot of log odd ratio (horizontal axis) and the standard error for the log odd ratio (vertical axis) for assessment of cough improvement in different areas after anti-reflux surgery.

**e-Figure 15** Funnel plot of log odd ratio (horizontal axis) and the standard error for the log odd ratio (vertical axis) for assessment of symptoms improvement after anti-reflux surgery.

**e-Figure 16** Funnel plot of log odd ratio (horizontal axis) and the standard error for the log odd ratio (vertical axis) for assessment of symptoms improvement after anti-reflux surgery.

**e-Figure 17** Funnel plot of log odd ratio (horizontal axis) and the standard error for the log odd ratio (vertical axis) for assessment of cough improvement after anti-reflux surgery.

**e-Table 1** Baseline characteristics of the study population

**e-Table 2** Meta-regression analysis for cough remission

**Included studies**

1. Ackroyd R, Watson DI, Devitt PG, Jamieson GG. Laparoscopic cardiomyotomy and anterior partial fundoplication for achalasia. Surgical endoscopy. Jul 2001;15(7):683-6. doi:10.1007/s004640080037

2. Adaba F, Ang CW, Perry A, Wadley MS, Robertson CS. Outcome of gastro-oesophageal reflux-related respiratory manifestations after laparoscopic fundoplication. International Journal of Surgery (London, England). 2014 2014;12(3):241-244. doi:10.1016/j.ijsu.2014.01.005

3. Aiolfi A, Cavalli M, Saino G, et al. Laparoscopic Toupet Fundoplication for the Treatment of Laryngopharyngeal Reflux: Results at Medium-Term follow-Up. World Journal of Surgery. 2020-11 2020;44(11):3821-3828. doi:10.1007/s00268-020-05653-5

4. Allen CJ, Anvari M. Gastro-oesophageal reflux related cough and its response to laparoscopic fundoplication. Thorax. 1998-11 1998;53(11):963-968. doi:10.1136/thx.53.11.963

5. Allen CJ, Anvari M. Preoperative symptom evaluation and esophageal acid infusion predict response to laparoscopic Nissen fundoplication in gastroesophageal reflux patients who present with cough. Surgical endoscopy. 2002-07 2002;16(7):1037-1041. doi:10.1007/s00464-001-8330-4

6. Allen CJ, Anvari M. Does laparoscopic fundoplication provide long-term control of gastroesophageal reflux related cough? Surgical endoscopy. 2004-04 2004;18(4):633-637. doi:10.1007/s00464-003-8821-6

7. Anvari M, Allen C, Moran LA. Immediate and delayed effects of laparoscopic Nissen fundoplication on pulmonary function. Surgical endoscopy. 1996-12 1996;10(12):1171-1175. doi:10.1007/s004649900272

8. Bott, E J, S P, Sw H. Proximal acid reflux treated by fundoplication predicts a good outcome for chronic cough attributable to gastro-oesophageal reflux disease. Langenbeck's archives of surgery. 2011 Feb 2011;396(2)doi:10.1007/s00423-010-0702-6

9. Brouwer R, Kiroff GK. Improvement of respiratory symptoms following laparoscopic Nissen fundoplication. ANZ journal of surgery. 2003-04 2003;73(4):189-193. doi:10.1046/j.1445-1433.2002.02568.x

10. Brown SR, Gyawali CP, Melman L, et al. Clinical outcomes of atypical extra-esophageal reflux symptoms following laparoscopic antireflux surgery. Surgical endoscopy. 2011-12 2011;25(12):3852-3858. doi:10.1007/s00464-011-1806-y

11. Chen D, Wang Z, Hu Z, Liang Y, Xiao F, Wu J. Typical symptoms and not positive reflux-cough correlation predict cure of gastroesophageal reflux disease related chronic cough after laparoscopic fundoplication: a retrospective study. BMC gastroenterology. 2019-06-26 2019;19(1):108. doi:10.1186/s12876-019-1027-8

12. Ciovica R, Gadenstätter M, Klingler A, Neumayer C, Schwab GP. Laparoscopic antireflux surgery provides excellent results and quality of life in gastroesophageal reflux disease patients with respiratory symptoms. Journal of Gastrointestinal Surgery: Official Journal of the Society for Surgery of the Alimentary Tract. 2005 May-Jun 2005;9(5):633-637. doi:10.1016/j.gassur.2005.02.008

13. Drews G, Rudolph F, Martinenko O, Kühne P, Schreiber J. [The Influence of Laparoscopic Fundoplication on Reflux-Associated Cough]. Zentralblatt Fur Chirurgie. 2016-10 2016;141(5):545-551. doi:10.1055/s-0034-1382899

14. Ekström T, Johansson KE. Effects of anti-reflux surgery on chronic cough and asthma in patients with gastro-oesophageal reflux disease. Respiratory Medicine. 2000-12 2000;94(12):1166-1170. doi:10.1053/rmed.2000.0944

15. Esposito C, Saxena A, Irtan S, Till H, Escolino M. Laparoscopic Nissen Fundoplication: An Excellent Treatment of GERD-Related Respiratory Symptoms in Children-Results of a Multicentric Study. Journal of Laparoendoscopic & Advanced Surgical Techniques Part A. 2018-08 2018;28(8):1023-1028. doi:10.1089/lap.2017.0631

16. Farrell TM, Richardson WS, Trus TL, Smith CD, Hunter JG. Response of atypical symptoms of gastro-oesophageal reflux to antireflux surgery. The British Journal of Surgery. 2001-12 2001;88(12):1649-1652. doi:10.1046/j.0007-1323.2001.01949.x

17. Frankel A, Ong HS, Smithers BM, Nathanson LK, Gotley DC. Efficacy of laparoscopic fundoplication in patients with chronic cough and gastro-oesophageal reflux. Esophagus. Jan 2023;20(1):170-177. doi:10.1007/s10388-022-00953-2

18. Gisi C, Wang K, Khan F, et al. Efficacy and patient satisfaction of single-session transoral incisionless fundoplication and laparoscopic hernia repair. Surgical endoscopy. Feb 2021;35(2):921-927. doi:10.1007/s00464-020-07796-x

19. Greason KL, Miller DL, Deschamps C, et al. Effects of antireflux procedures on respiratory symptoms. The Annals of Thoracic Surgery. 2002-02 2002;73(2):381-385. doi:10.1016/s0003-4975(01)03407-5

20. Herbella FA, Andolfi C, Vigneswaran Y, Patti MG, Pinna BR. Importance of esophageal manometry and pH monitoring for the evaluation of otorhinolaryngologic (ENT) manifestations of GERD. A multicenter study. J Gastrointest Surg. Oct 2016;20(10):1673-8. doi:10.1007/s11605-016-3212-1

21. Hoppo T, Komatsu Y, Jobe BA. Antireflux surgery in patients with chronic cough and abnormal proximal exposure as measured by hypopharyngeal multichannel intraluminal impedance. JAMA surgery. 2013-07 2013;148(7):608-615. doi:10.1001/jamasurg.2013.1376

22. Hui TT, Fass SM, Giurgiu DI, Iida A, Takagi S, Phillips EH. Gastroesophageal disease and nausea: does fundoplication help or hurt? Arch Surg. May 2000;135(5):545-9. doi:10.1001/archsurg.135.5.545

23. Hunter JG, Trus TL, Branum GD, Waring JP, Wood WC. A physiologic approach to laparoscopic fundoplication for gastroesophageal reflux disease. Annals of Surgery. 1996-06 1996;223(6):673-685; discussion 685-687. doi:10.1097/00000658-199606000-00006

24. Iqbal M, Batch AJ, Moorthy K, Cooper BT, Spychal RT. Outcome of surgical fundoplication for extra-oesophageal symptoms of reflux. Surgical endoscopy. 2009-03 2009;23(3):557-561. doi:10.1007/s00464-008-9861-8

25. Irwin, Jk Z, Mm W, Ct F, Mp C. Chronic cough due to gastroesophageal reflux disease: failure to resolve despite total/near-total elimination of esophageal acid. Chest. 2002 Apr 2002;121(4)doi:10.1378/chest.121.4.1132

26. Johnson WE, Hagen JA, DeMeester TR, et al. Outcome of respiratory symptoms after antireflux surgery on patients with gastroesophageal reflux disease. Arch Surg. May 1996;131(5):489-92. doi:10.1001/archsurg.1996.01430170035005

27. Kalapala R, Shah H, Nabi Z, Darisetty S, Talukdar R, Nageshwar Reddy D. Treatment of gastroesophageal reflux disease using radiofrequency ablation (Stretta procedure): An interim analysis of a randomized trial. Indian Journal of Gastroenterology: Official Journal of the Indian Society of Gastroenterology. 2017-09 2017;36(5):337-342. doi:10.1007/s12664-017-0796-7

28. Kaufman JA, Houghland JE, Quiroga E, Cahill M, Pellegrini CA, Oelschlager BK. Long-term outcomes of laparoscopic antireflux surgery for gastroesophageal reflux disease (GERD)-related airway disorder. Surgical endoscopy. 2006-12 2006;20(12):1824-1830. doi:10.1007/s00464-005-0329-9

29. Kiljander T, Rantanen T, Kellokumpu I, et al. Comparison of the effects of esomeprazole and fundoplication on airway responsiveness in patients with gastro-oesophageal reflux disease. The Clinical Respiratory Journal. 2013-07 2013;7(3):281-287. doi:10.1111/crj.12005

30. Koch OO, Antoniou SA, Kaindlstorfer A, Asche KU, Granderath FA, Pointner R. Effectiveness of laparoscopic total and partial fundoplication on extraesophageal manifestations of gastroesophageal reflux disease: a randomized study. Surgical Laparoscopy, Endoscopy & Percutaneous Techniques. 2012-10 2012;22(5):387-391. doi:10.1097/SLE.0b013e31825efb5b

31. Liang W-T, Wang Z-G, Wang F, et al. Long-term outcomes of patients with refractory gastroesophageal reflux disease following a minimally invasive endoscopic procedure: a prospective observational study. BMC gastroenterology. 2014-10-10 2014;14:178. doi:10.1186/1471-230X-14-178

32. Liang W-T, Yan C, Wang Z-G, et al. Early and Midterm Outcome After Laparoscopic Fundoplication and a Minimally Invasive Endoscopic Procedure in Patients with Gastroesophageal Reflux Disease: A Prospective Observational Study. Journal of Laparoendoscopic & Advanced Surgical Techniques Part A. 2015-08 2015;25(8):657-661. doi:10.1089/lap.2015.0188

33. Liang WT, Wu JM, Wang F, Hu ZW, Wang ZG. Stretta radiofrequency for gastroesophageal reflux disease-related respiratory symptoms: a prospective 5-year study. Minerva Chirurgica. 2014-10 2014;69(5):293-299.

34. Lindstrom DR, Wallace J, Loehrl TA, Merati AL, Toohill RJ. Nissen fundoplication surgery for extraesophageal manifestations of gastroesophageal reflux (EER). The Laryngoscope. 2002-10 2002;112(10):1762-1765. doi:10.1097/00005537-200210000-00010

35. Liu JJ, Carr-Locke DL, Osterman MT, et al. Endoscopic treatment for atypical manifestations of gastroesophageal reflux disease. The American journal of gastroenterology. 2006-03 2006;101(3):440-445. doi:10.1111/j.1572-0241.2006.00496.x

36. Lomasney TL. Hiatus hernia and the respiratory tract. The Annals of Thoracic Surgery. 1977-11 1977;24(5):448-450. doi:10.1016/s0003-4975(10)63439-x

37. Lugaresi M, Aramini B, Daddi N, Baldi F, Mattioli S. Effectiveness of antireflux surgery for the cure of chronic cough associated with gastroesophageal reflux disease. World Journal of Surgery. 2015-01 2015;39(1):208-215. doi:10.1007/s00268-014-2769-7

38. McClusky DA, Khaitan L, Swafford VA, Smith CD. Radiofrequency energy delivery to the lower esophageal sphincter (Stretta procedure) in patients with recurrent reflux after antireflux surgery: can surgery be avoided? Surgical endoscopy. 2007-07 2007;21(7):1207-1211. doi:10.1007/s00464-007-9195-y

39. Novitsky YW, Zawacki JK, Irwin RS, French CT, Hussey VM, Callery MP. Chronic cough due to gastroesophageal reflux disease: efficacy of antireflux surgery. Surgical endoscopy. 2002-04 2002;16(4):567-571. doi:10.1007/s00464-001-8328-y

40. Oelschlager BK, Eubanks TR, Oleynikov D, Pope C, Pellegrini CA. Symptomatic and physiologic outcomes after operative treatment for extraesophageal reflux. Surgical endoscopy. 2002-07 2002;16(7):1032-1036. doi:10.1007/s00464-001-8252-1

41. Oelschlager BK, Quiroga E, Parra JD, Cahill M, Polissar N, Pellegrini CA. Long-term outcomes after laparoscopic antireflux surgery. The American journal of gastroenterology. 2008-02 2008;103(2):280-287; quiz 288. doi:10.1111/j.1572-0241.2007.01606.x

42. Park A, Weltz AS, Sanford Z, Addo A, Zahiri HR. Laparoscopic antireflux surgery (LARS) is highly effective in the treatment of select patients with chronic cough. Surgery. 2019-07 2019;166(1):34-40. doi:10.1016/j.surg.2019.01.036

43. Patti MG. Antireflux surgery, a cough medicine difficult to swallow for most physicians: Comment on "Antireflux surgery in patients with chronic cough and abnormal proximal exposure as measured by hypopharyngeal multichannel intraluminal impedance". JAMA surgery. 2013-07 2013;148(7):615-616. doi:10.1001/jamasurg.2013.1387

44. Patti MG, Arcerito M, Tamburini A, et al. Effect of laparoscopic fundoplication on gastroesophageal reflux disease-induced respiratory symptoms. Journal of Gastrointestinal Surgery: Official Journal of the Society for Surgery of the Alimentary Tract. 2000 Mar-Apr 2000;4(2):143-149. doi:10.1016/s1091-255x(00)80050-5

45. Ranson ME, Danielson A, Maxwell JG, Harris JA. Prospective study of laparoscopic nissen fundoplication in a community hospital and its effect on typical, atypical, and nonspecific gastrointestinal symptoms. JSLS: Journal of the Society of Laparoendoscopic Surgeons. 2007 Jan-Mar 2007;11(1):66-71.

46. Ribet M, Pruvot FR, Mensier E, Ghoch K, Rousseau B. Gastro-oesophageal reflux and respiratory disorders treated by Hill's procedure. European Journal of Cardio-Thoracic Surgery: Official Journal of the European Association for Cardio-Thoracic Surgery. 1989 1989;3(5):414-417; discussion 418. doi:10.1016/1010-7940(89)90050-x

47. Swoger J, Ponsky J, Hicks DM, et al. Surgical fundoplication in laryngopharyngeal reflux unresponsive to aggressive acid suppression: a controlled study. Clinical Gastroenterology and Hepatology: The Official Clinical Practice Journal of the American Gastroenterological Association. 2006-04 2006;4(4):433-441. doi:10.1016/j.cgh.2006.01.011

48. Thoman DS, Hui TT, Spyrou M, Phillips EH. Laparoscopic antireflux surgery and its effect on cough in patients with gastroesophageal reflux disease. Journal of Gastrointestinal Surgery: Official Journal of the Society for Surgery of the Alimentary Tract. 2002 Jan-Feb 2002;6(1):17-21. doi:10.1016/s1091-255x(01)00013-0

49. Tibbling L. Wrong-way swallowing as a possible cause of bronchitis in patients with gastroesophageal reflux disease. Acta Otolaryngol. May 1993;113(3):405-8. doi:10.3109/00016489309135835

50. Toomey P, Teta A, Patel K, Ross S, Sukharamwala P, Rosemurgy AS. Transoral incisionless fundoplication: is it as safe and efficacious as a Nissen or Toupet fundoplication? The American Surgeon. 2014-09 2014;80(9):860-867.

51. Trad KS, Turgeon DG, Deljkich E. Long-term outcomes after transoral incisionless fundoplication in patients with GERD and LPR symptoms. Surgical endoscopy. 2012-03 2012;26(3):650-660. doi:10.1007/s00464-011-1932-6

52. van der Westhuizen L, Von SJ, Wilkerson BJ, et al. Impact of Nissen fundoplication on laryngopharyngeal reflux symptoms. The American Surgeon. 2011-07 2011;77(7):878-882. doi:10.1177/000313481107700723

53. Wassenaar E, Johnston N, Merati A, et al. Pepsin detection in patients with laryngopharyngeal reflux before and after fundoplication. Surgical endoscopy. 2011-12 2011;25(12):3870-3876. doi:10.1007/s00464-011-1813-z

54. Wetscher

GJ, Glaser K, Hinder RA, et al. Respiratory symptoms in patients with gastroesophageal reflux disease following medical therapy and following antireflux surgery. American Journal of Surgery. 1997-12 1997;174(6):639-642; discussion 642-643. doi:10.1016/s0002-9610(97)00180-3

55. White B, Jeansonne LO, Cook M, et al. Use of endoluminal antireflux therapies for obese patients with GERD. Obesity Surgery. 2009-06 2009;19(6):783-787. doi:10.1007/s11695-008-9715-4

56. Wright RC, Rhodes KP. Improvement of laryngopharyngeal reflux symptoms after laparoscopic Hill repair. American Journal of Surgery. 2003-05 2003;185(5):455-461. doi:10.1016/s0002-9610(03)00052-7

57. Yan C, Liang W-T, Wang Z-G, et al. Comparison of Stretta procedure and toupet fundoplication for gastroesophageal reflux disease-related extra-esophageal symptoms. World Journal of Gastroenterology. 2015-12-07 2015;21(45):12882-12887. doi:10.3748/wjg.v21.i45.12882

58. Zhang C, Wang Z-g, Wu J-m, et al. A preliminary investigation of laparoscopic fundoplication treatment on gastroesophageal reflux disease-related respiratory symptoms. Surgical Laparoscopy, Endoscopy & Percutaneous Techniques. 2012-10 2012;22(5):406-409. doi:10.1097/SLE.0b013e3182628913

59. Ziora D, Jarosz W, Dzielicki J, et al. Citric acid cough threshold in patients with gastroesophageal reflux disease rises after laparoscopic fundoplication. Chest. 2005-10 2005;128(4):2458-2464. doi:10.1378/chest.128.4.2458


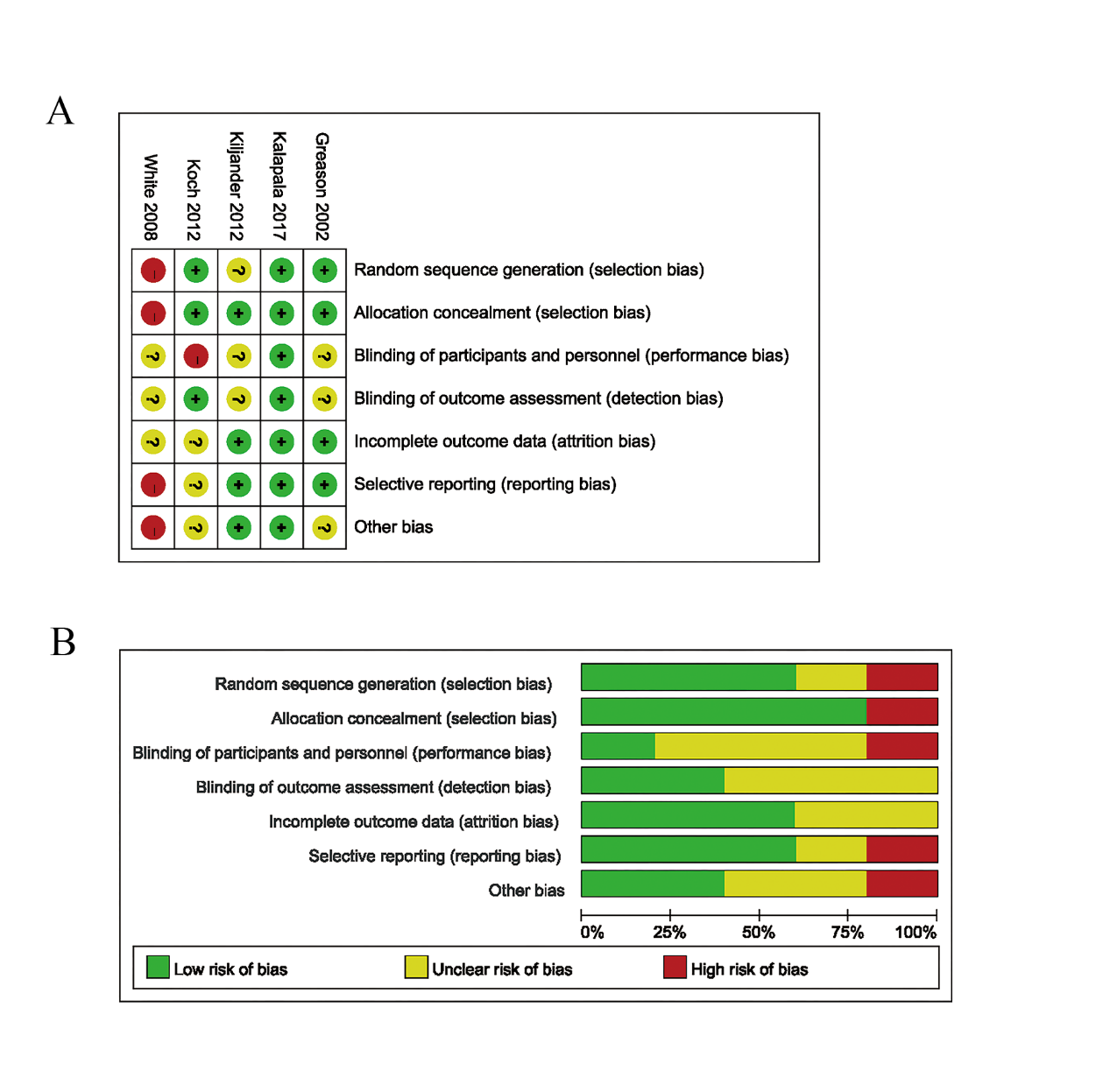


**e-Figure 1.** The results of the Cochrane tool for assessing the risk of bias. (A) Risk of bias graph. (B) Risk of bias summary. Colour of each circle shows different risk of bias. Red circle reflects high risk of bias, yellow reflects unclear risk of bias, and green reflects low risk of bias.


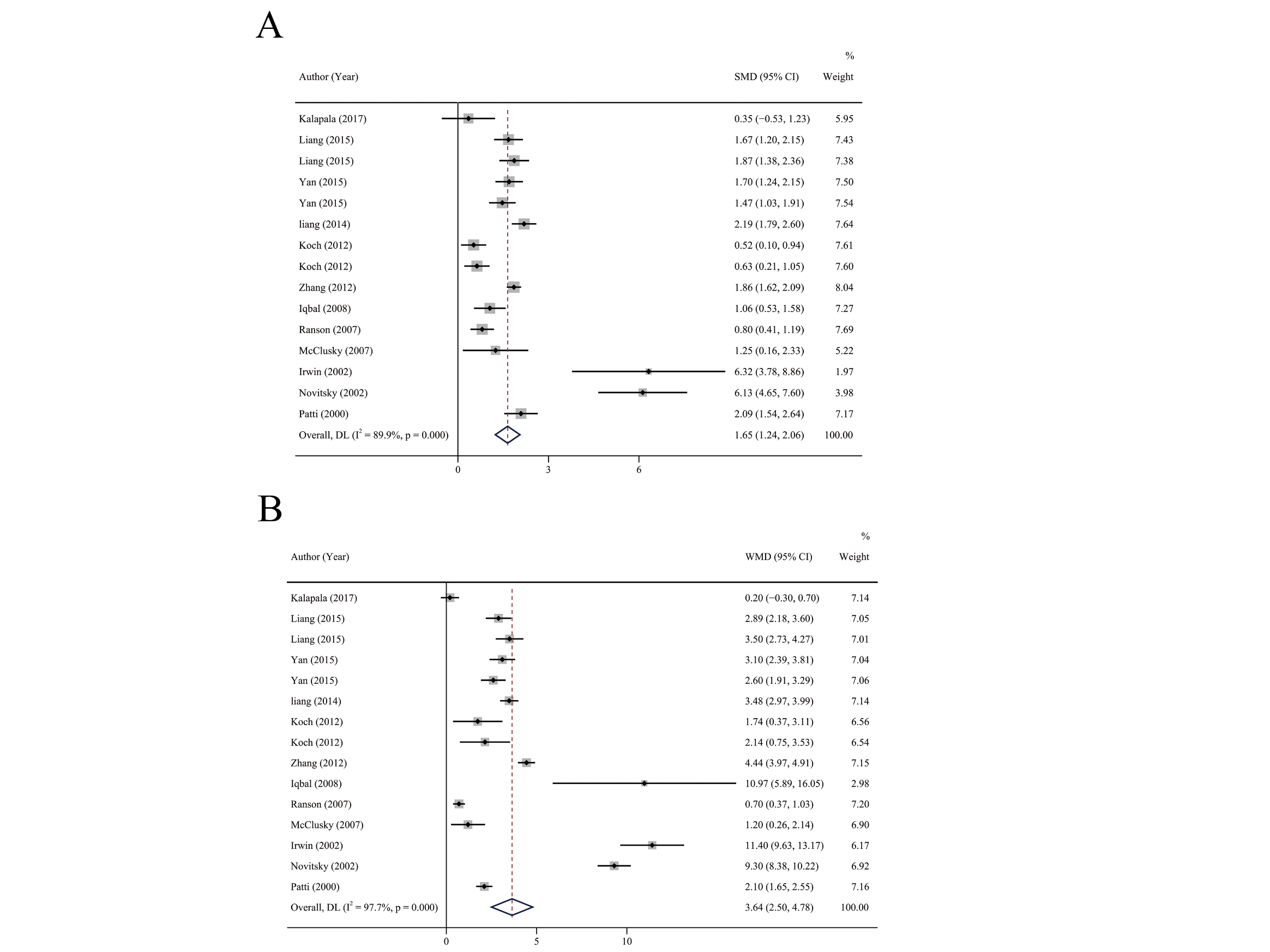


**e-Figure 2.** Forest plots of assessment of cough improvement after anti-reflux surgery. (A) Standardized mean difference of improvement in cough score. (B) Weighted mean difference of improvement in cough score.


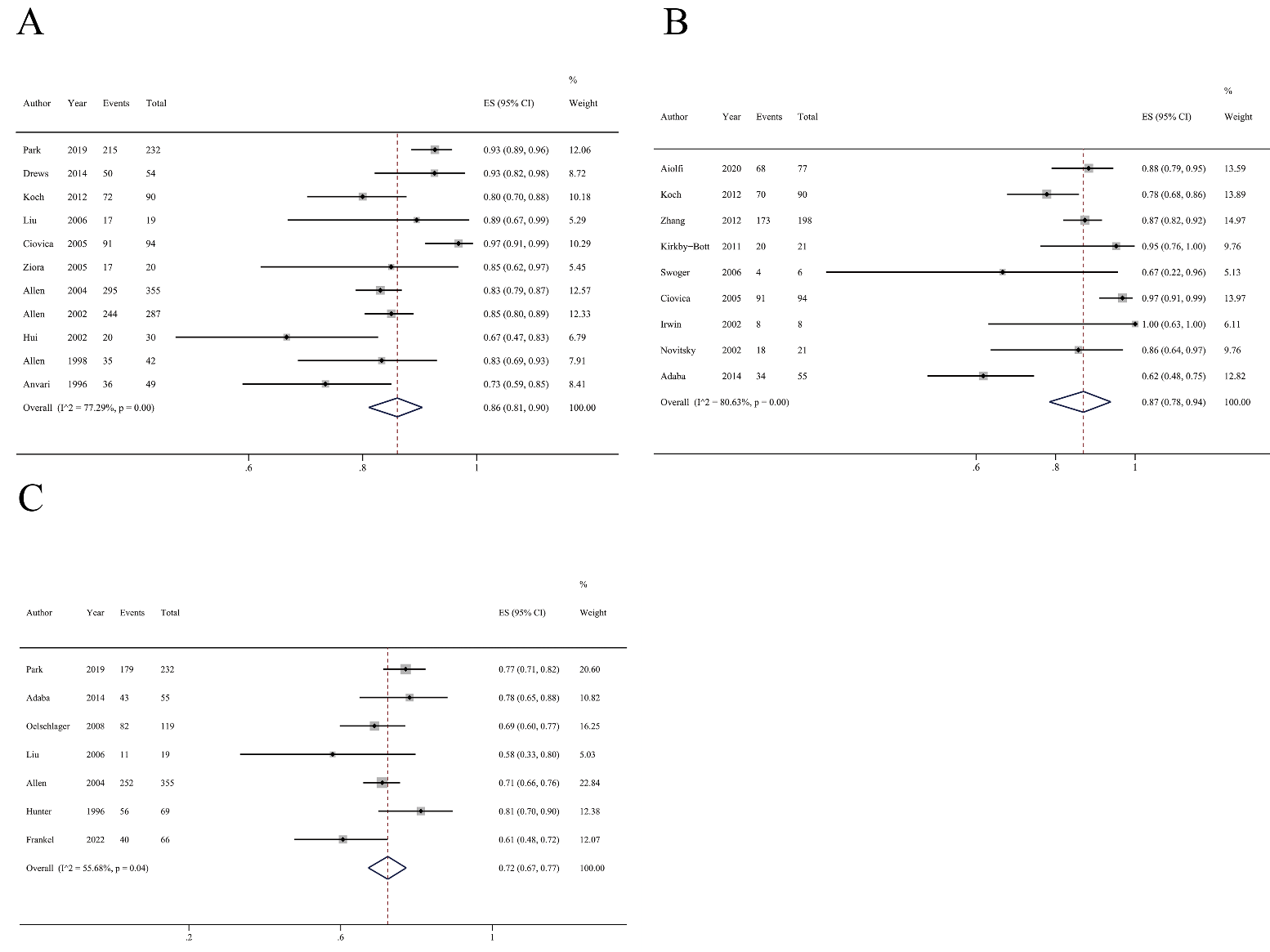


**e-Figure 3.** Forest plots of assessment of cough improvement in different follow-up time after anti-reflux surgery. (A)Remission rate of cough in less than 6 months. (B) Remission rate of cough between 6 and 12 months. (C) Remission rate of cough in more than12 months.


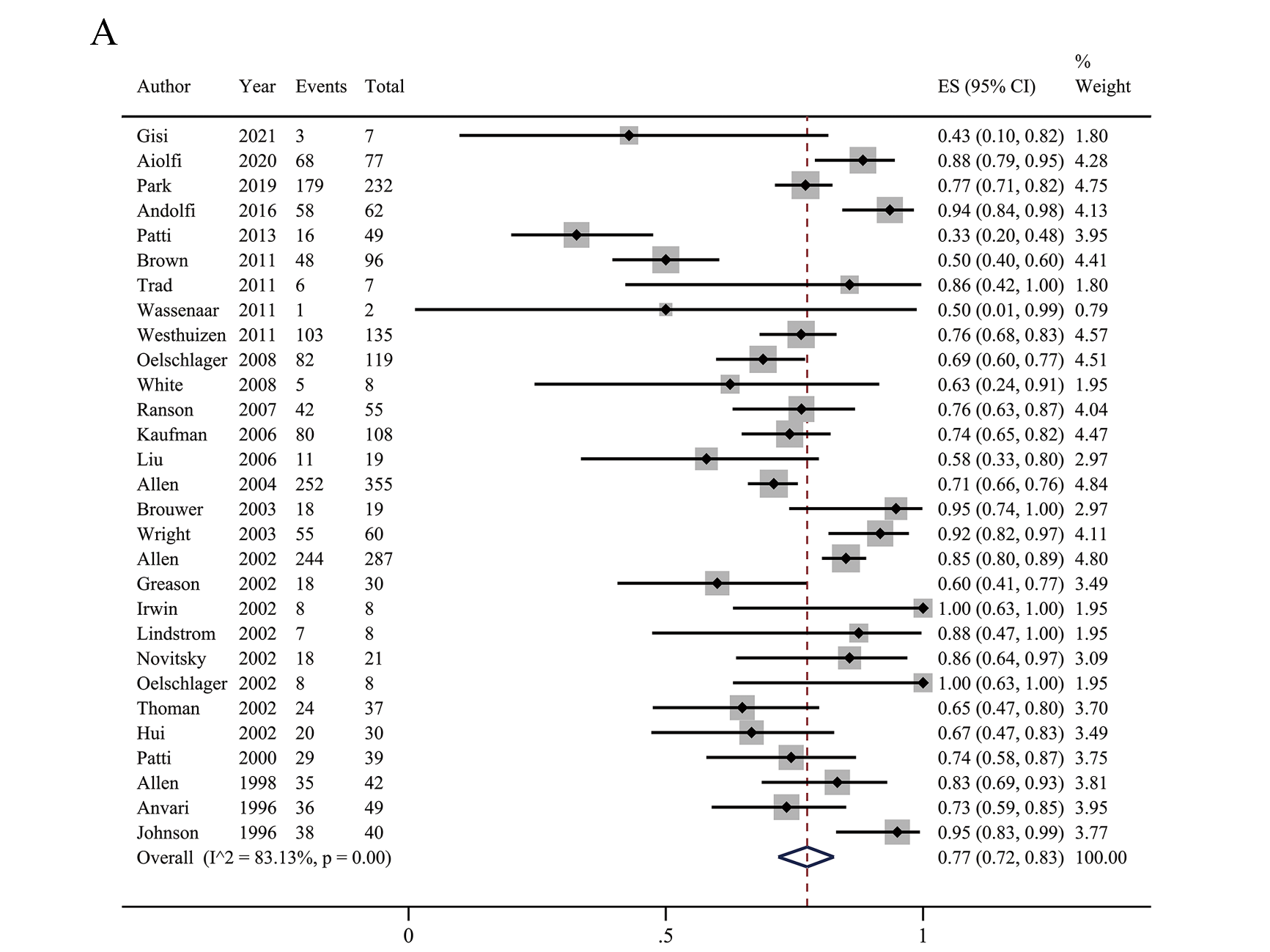
**e-Figure 4.** Forest plots of assessment of cough improvement in North America after anti-reflux surgery.


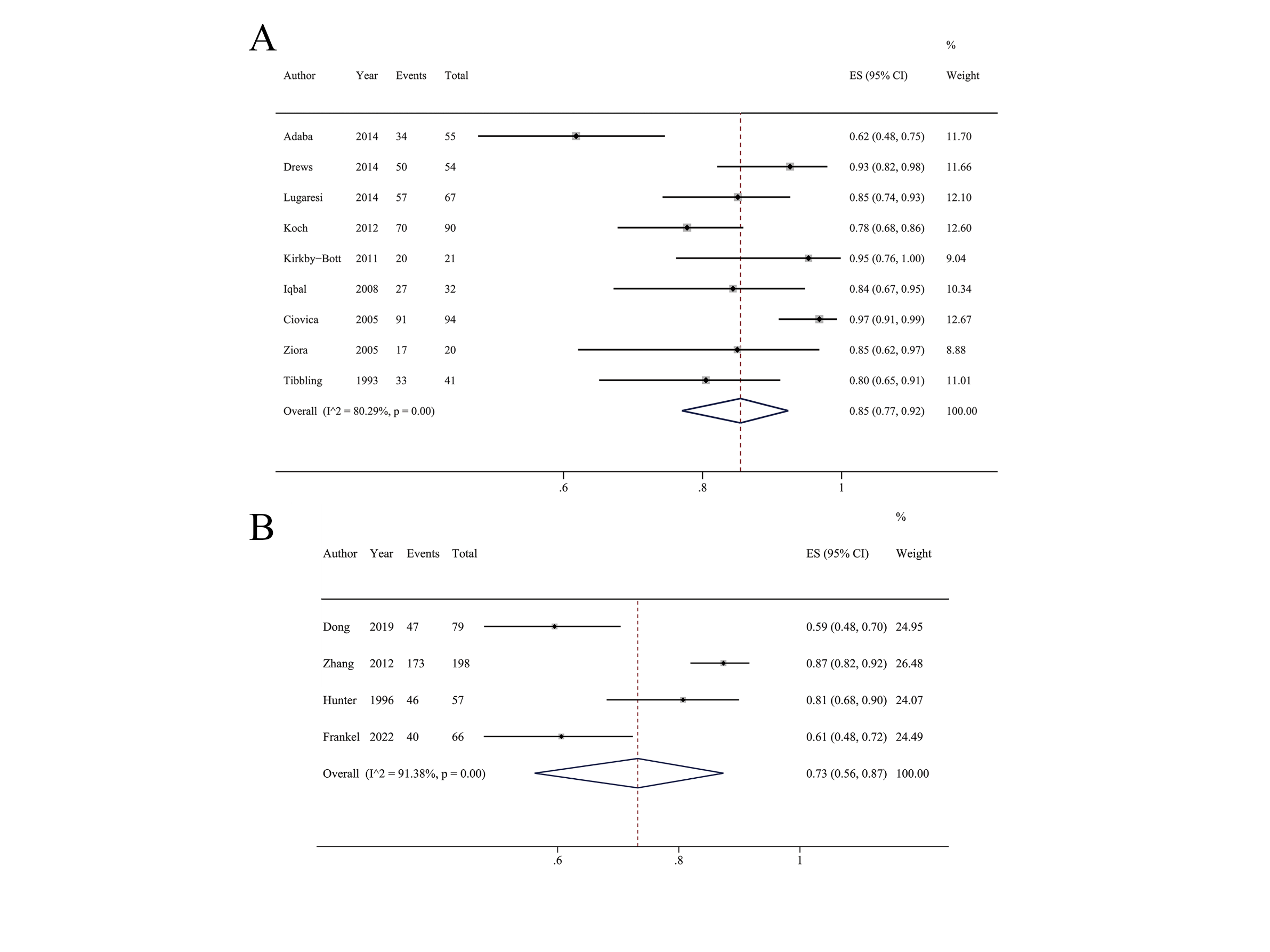


**e-Figure 5.** Forest plots of assessment of cough improvement in different areas after anti-reflux surgery. (A) Remission rate of cough in Europe. (B) Remission rate of cough in Asia.


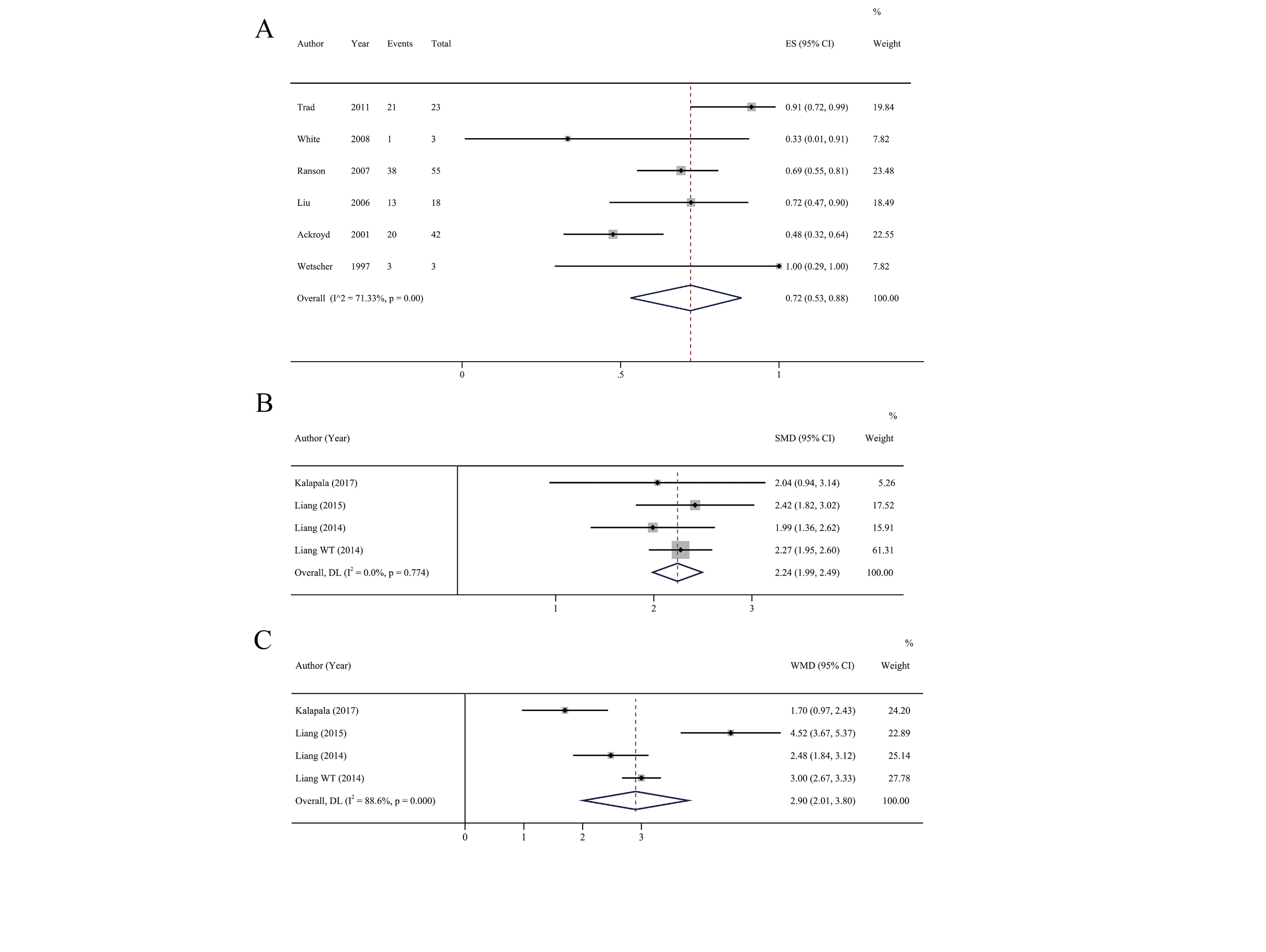


**e-Figure 6.** Forest plots of the assessment of chest pain improvement after anti-reflux surgery. (A)Remission rate of chest pain. (B) Standardized mean difference of improvement in chest pain score. (C) Weighted mean difference of improvement in chest pain score.


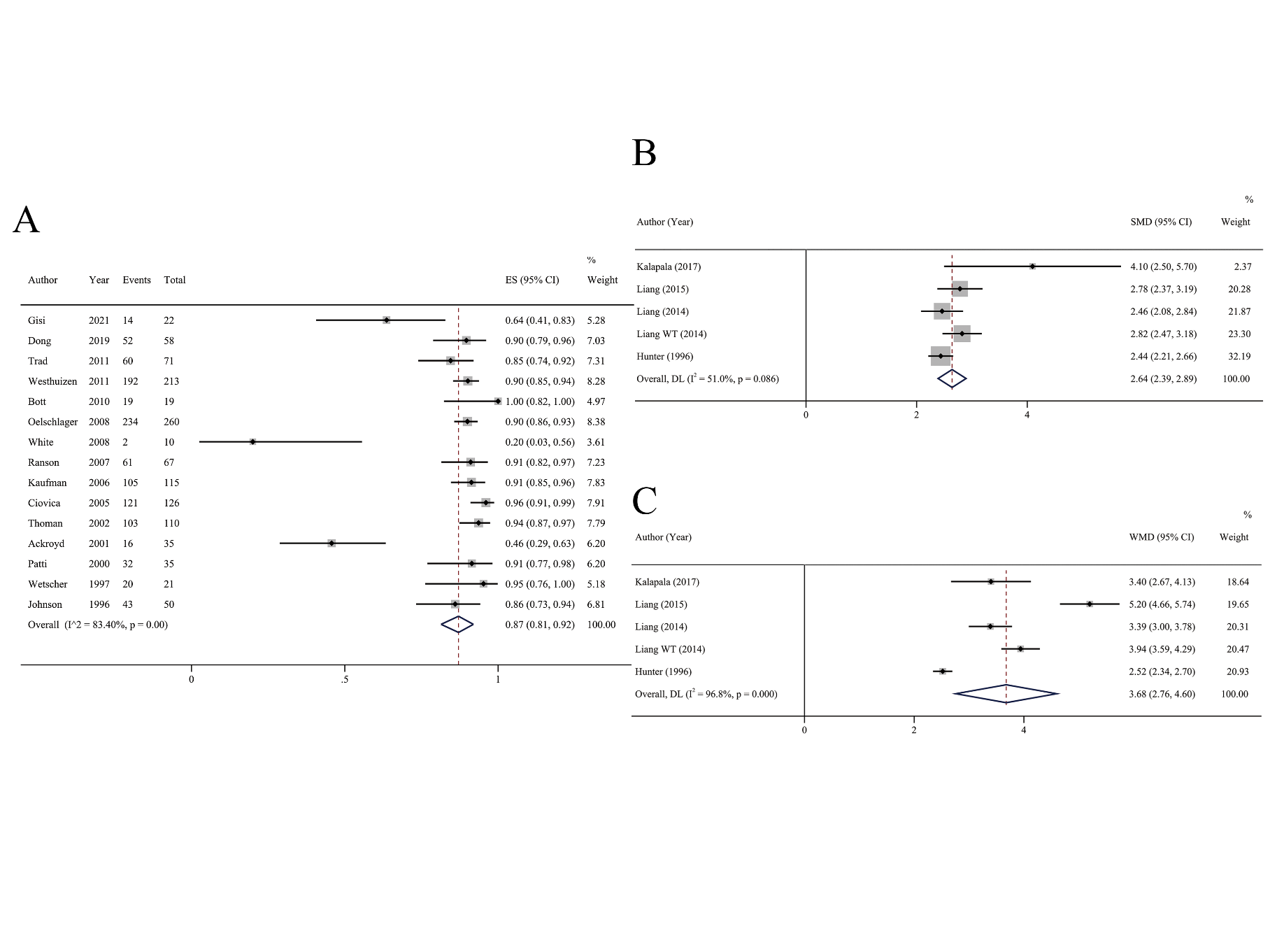


**e-Figure 7.** Forest plots of the assessment of heartburn improvement after anti-reflux surgery. (A)Remission rate of heartburn. (B) Standardized mean difference of improvement in heartburn score. (C) Weighted mean difference of improvement in heartburn score.


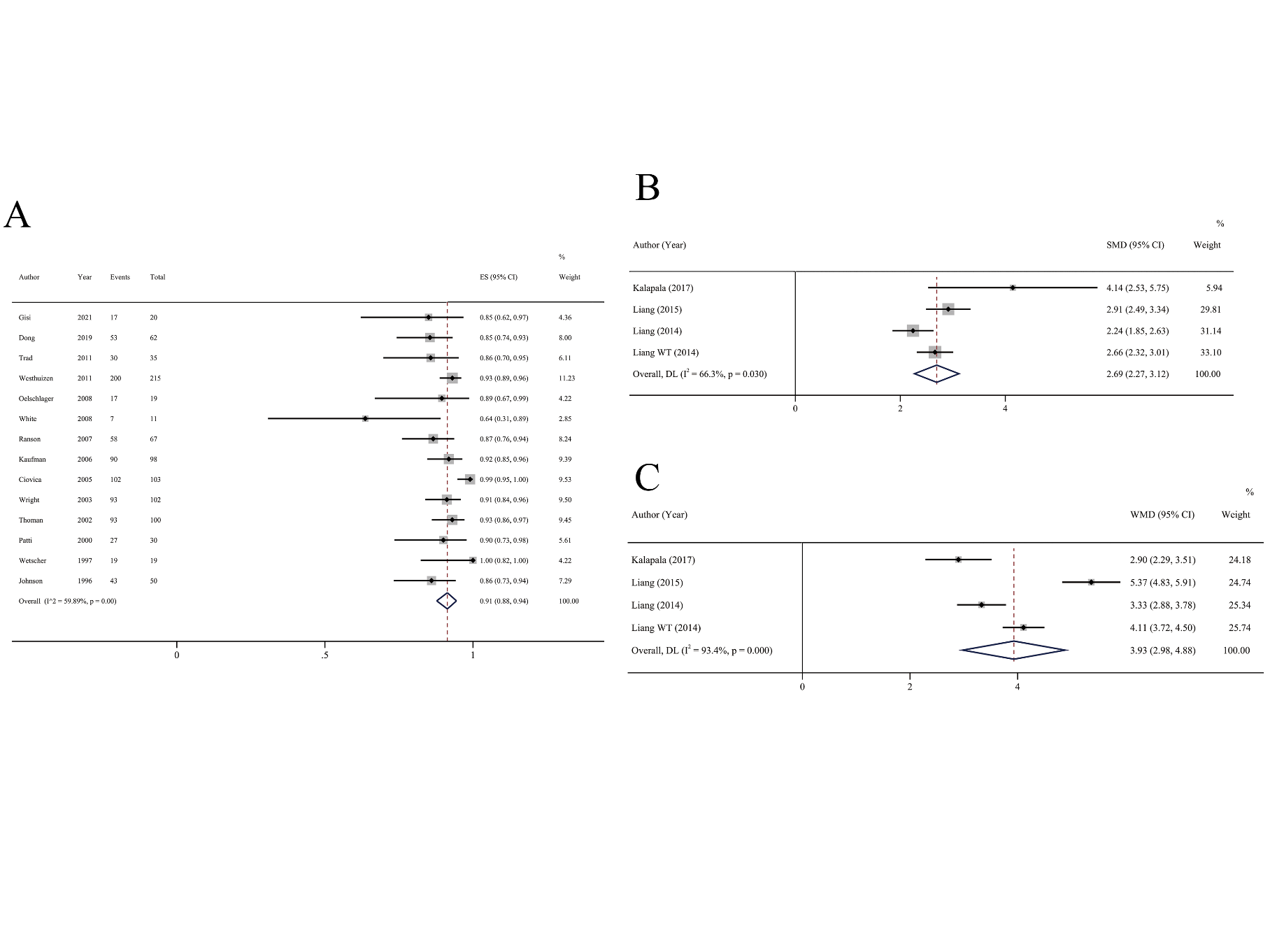


**e-Figure 8.** Forest plots of the assessment of regurgitation improvement after anti-reflux surgery. (A)Remission rate of regurgitation. (B) Standardized mean difference of improvement in regurgitation score. (C) Weighted mean difference of improvement in regurgitation score.


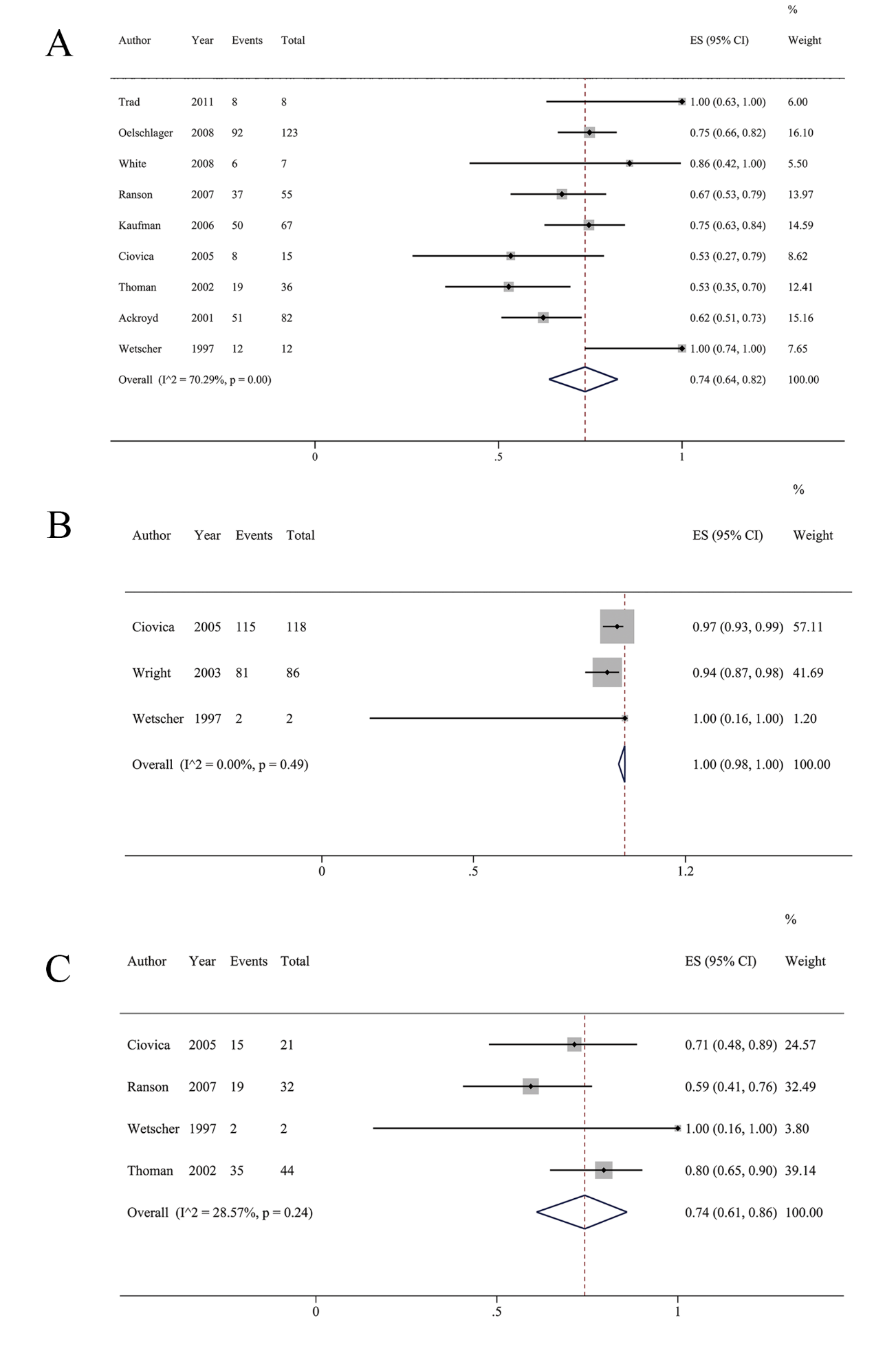


**e-Figure 9.** Forest plots of assessment of different symptoms improvement after anti-reflux surgery. (A) Remission rate of dysphagia.(B) Remission rate of epigastric pain. (C) Remission rate of nausea.


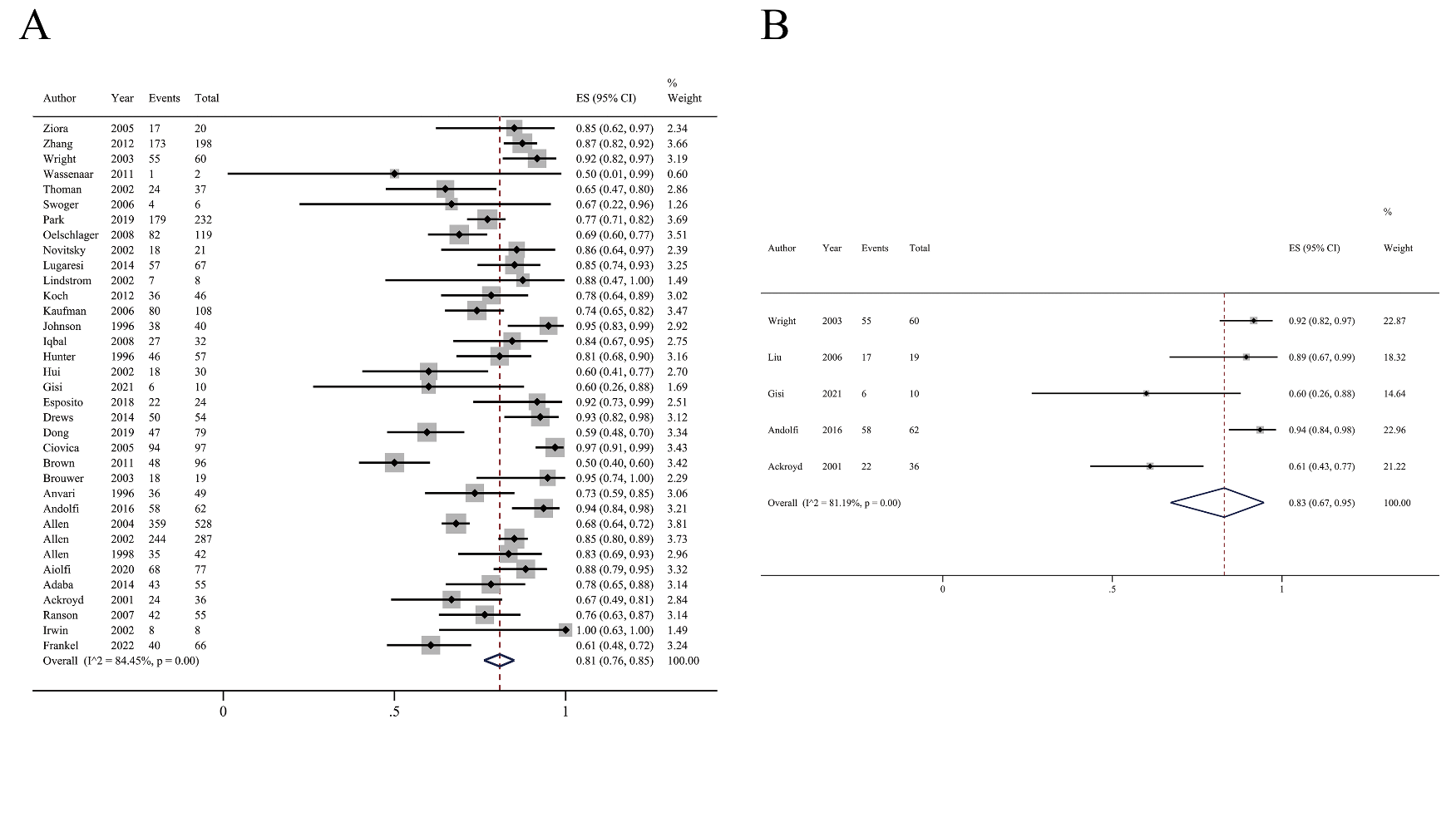


**e-Figure 10**. Forest plots of the association between the surgical techniques of anti-reflux surgery and the improvement of cough score in patients. (A)Nissen. (B) Other techniques.


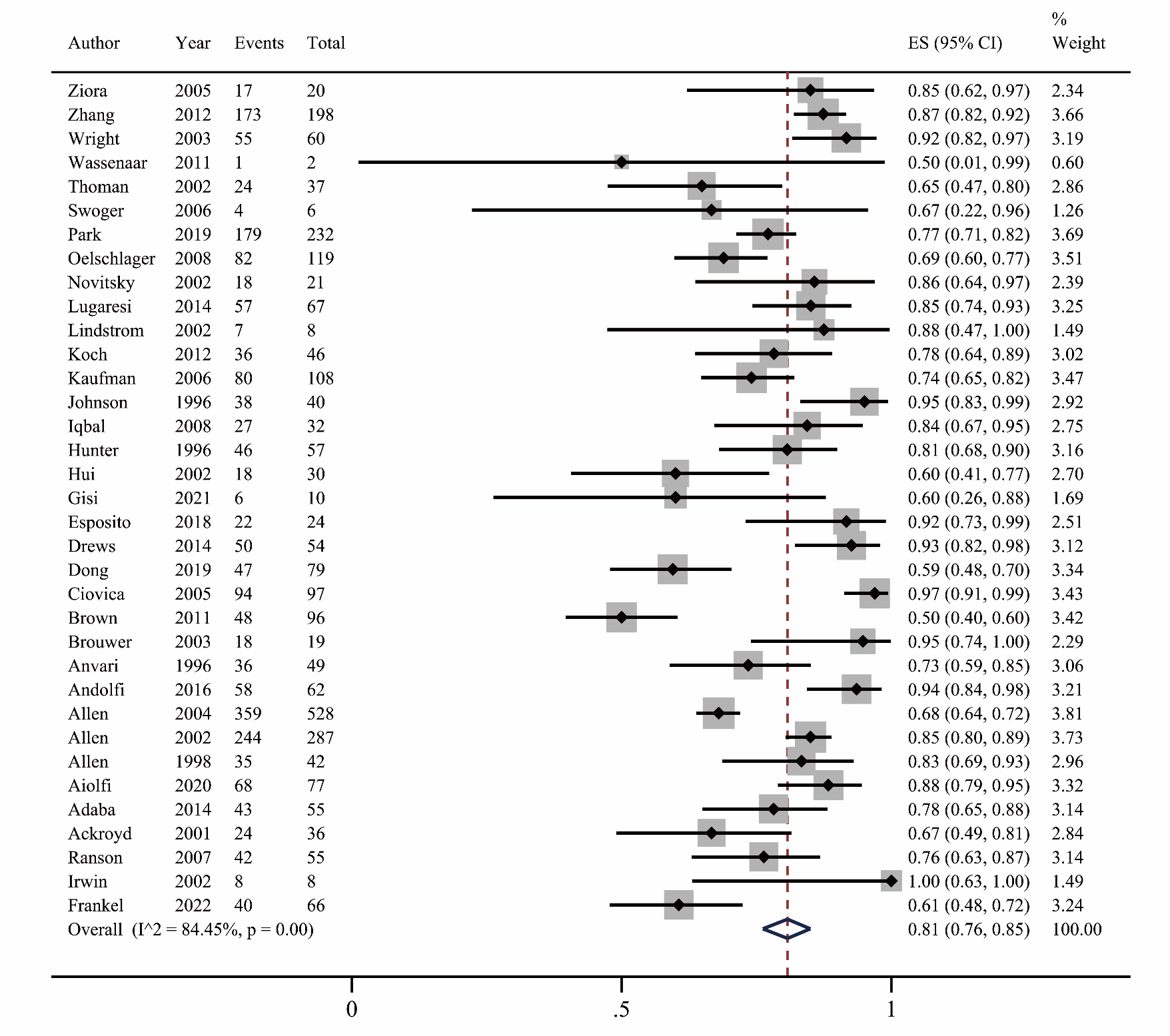


**e-Figure 11**. Forest plots of the improvement of cough score in patients who underwent laparoscopic surgery


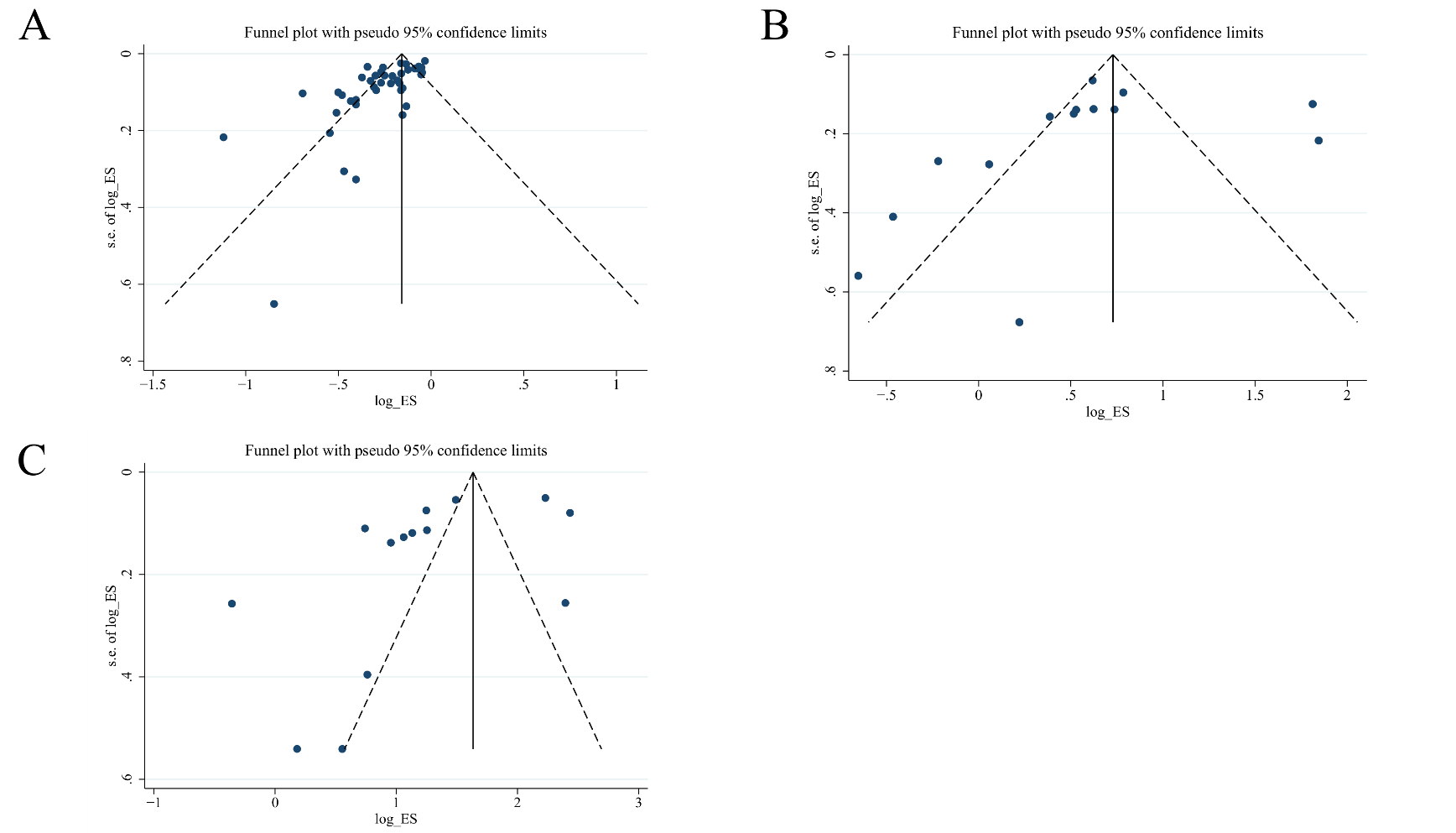


**e-Figure 12** Funnel plot of log odd ratio (horizontal axis) and the standard error for the log odd ratio (vertical axis) for assessment of cough improvement after anti-reflux surgery. (A) Remission rate of cough.(B) Weighted mean difference of improvement in cough score. (C) Standardized mean difference of improvement in cough score.


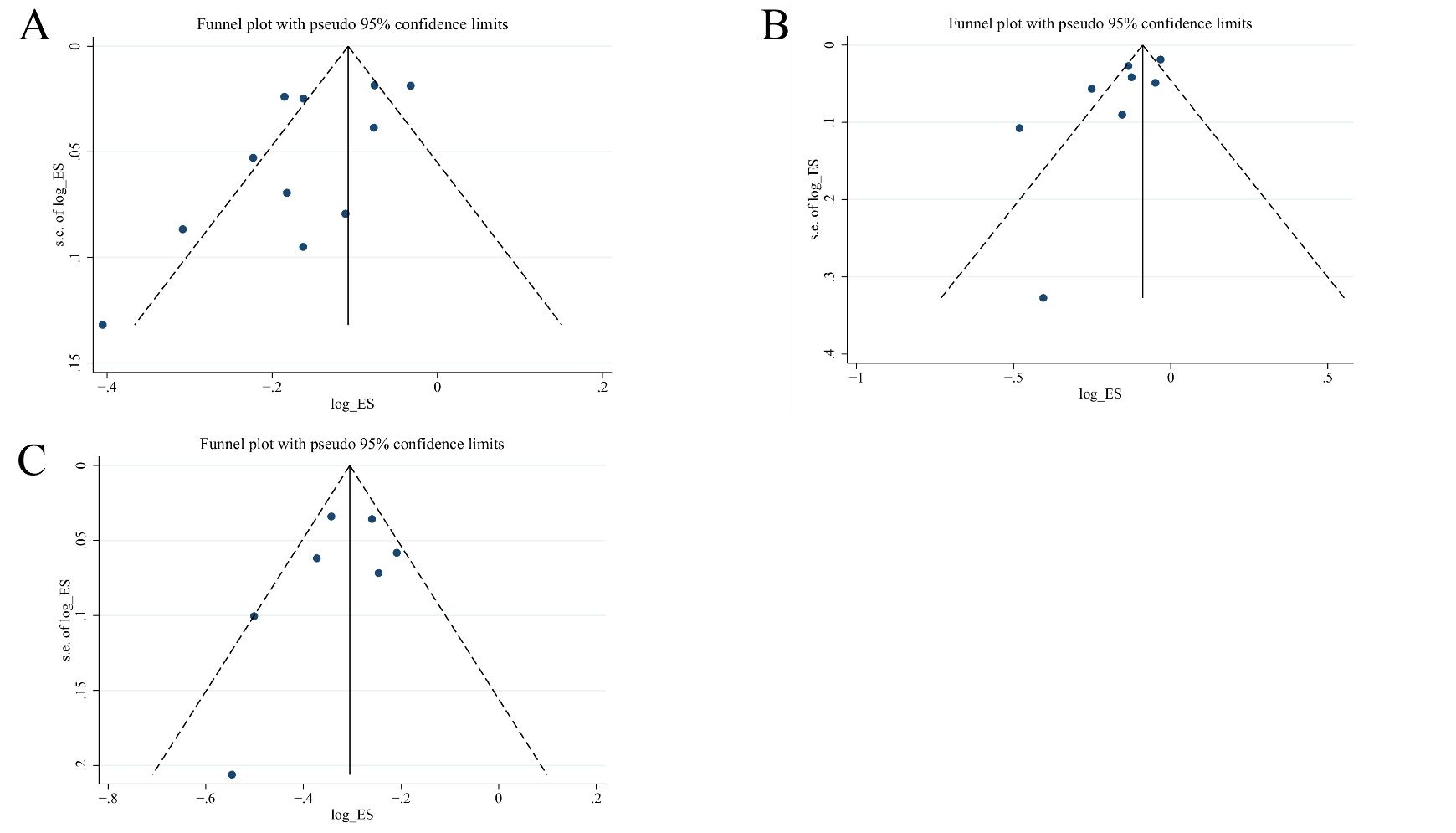


**e-Figure 13.** Funnel plot of log odd ratio (horizontal axis) and the standard error for the log odd ratio (vertical axis) for assessment of cough improvement in different follow-up time after anti-reflux surgery. (A)Remission rate of cough in less than 6 months. (B) Remission rate of cough between 6 and 12months. (C) Remission rate of cough in more than 12 months.


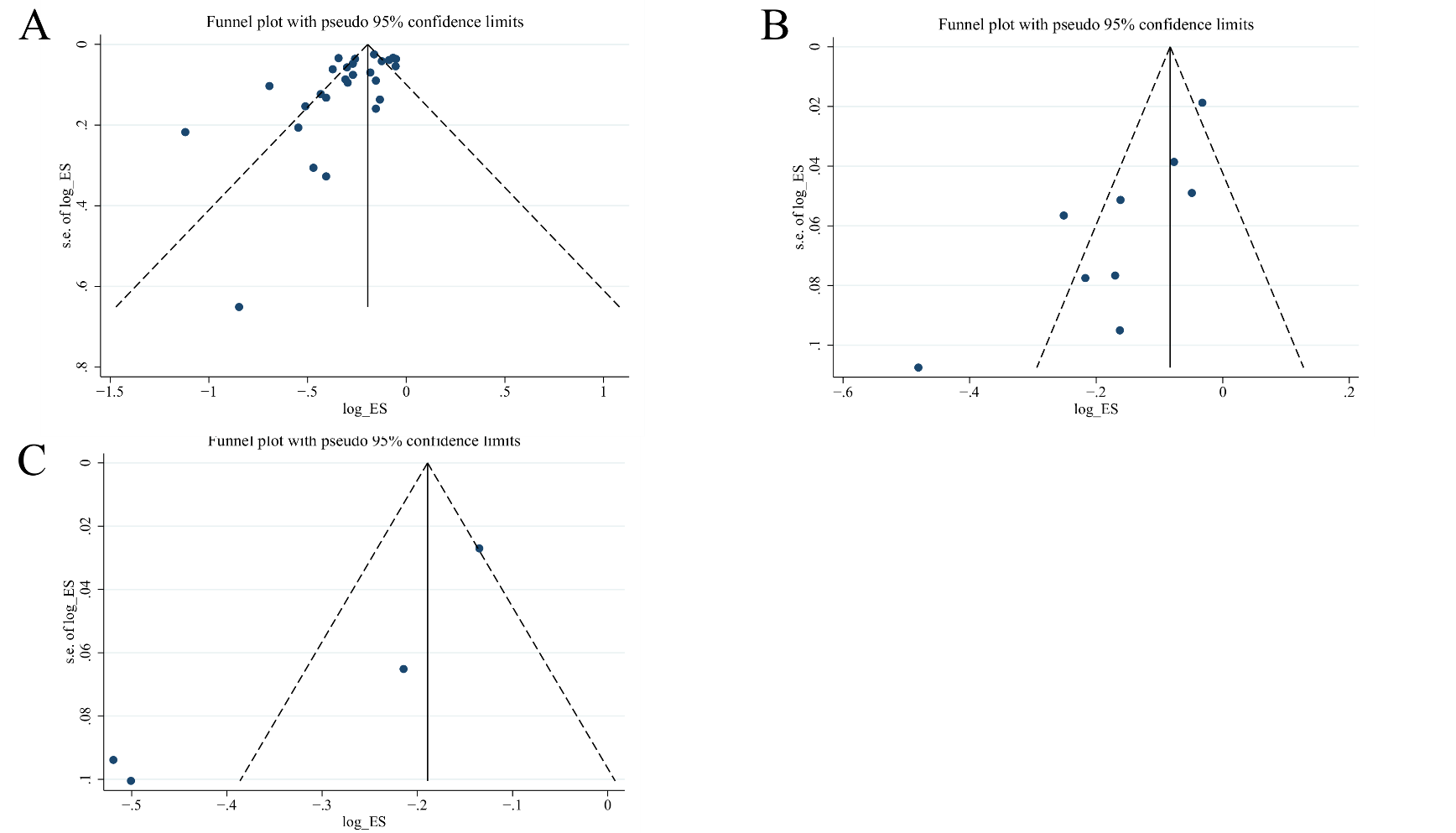


**e-Figure 13.** Funnel plot of log odd ratio (horizontal axis) and the standard error for the log odd ratio (vertical axis) for assessment of cough improvement in different areas after anti-reflux surgery. (A)Remission rate of cough in North America. (B) Remission rate of cough in Europe.(C) Remission rate of cough in Asia.

.


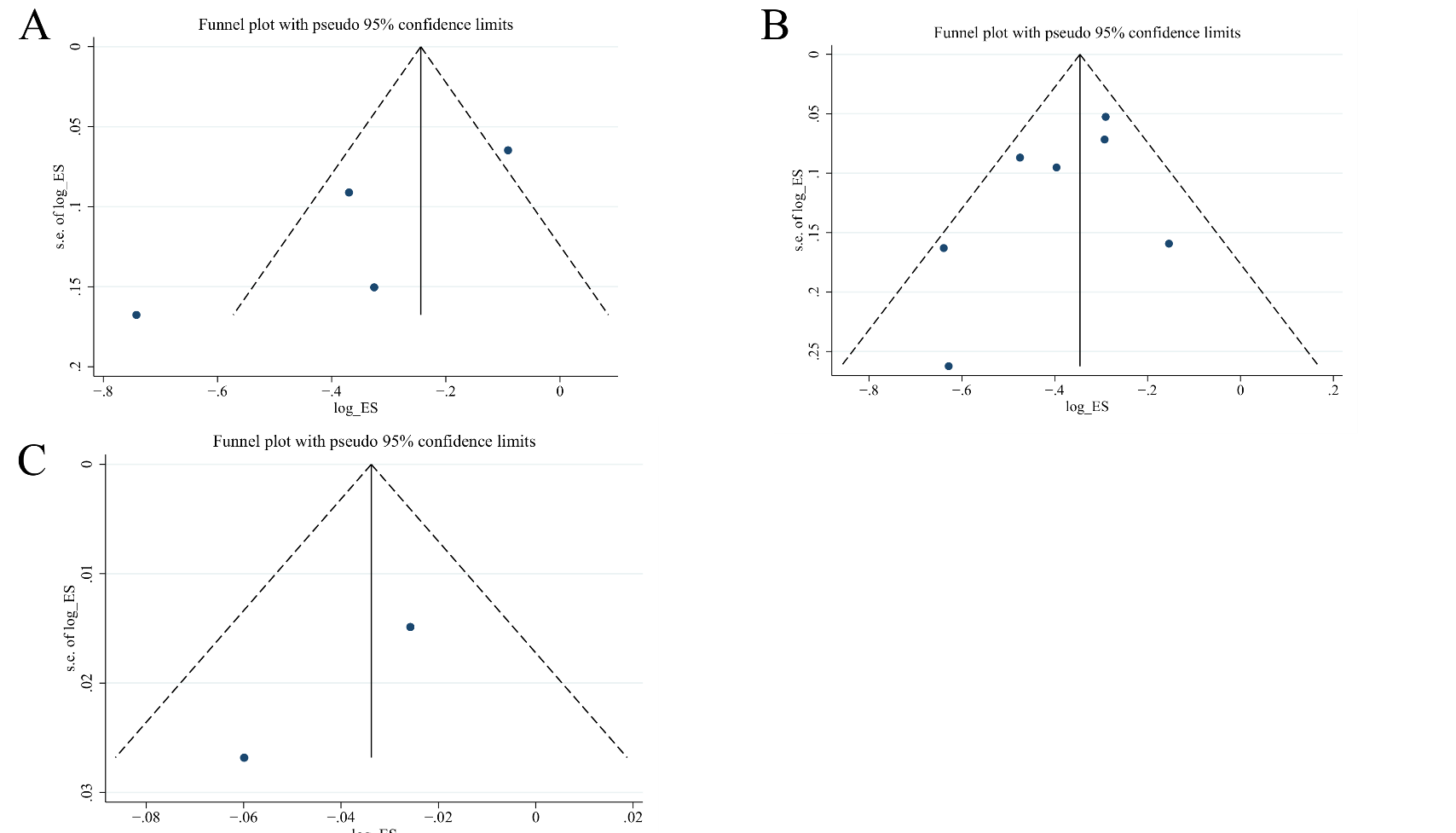


**e-Figure 15.** Funnel plot of log odd ratio (horizontal axis) and the standard error for the log odd ratio (vertical axis) for assessment of symptoms improvement after anti-reflux surgery. (A) Remission rate of chest pain. (B) Remission rate of dysphagia. (C)Remission rate of epigastric pain.


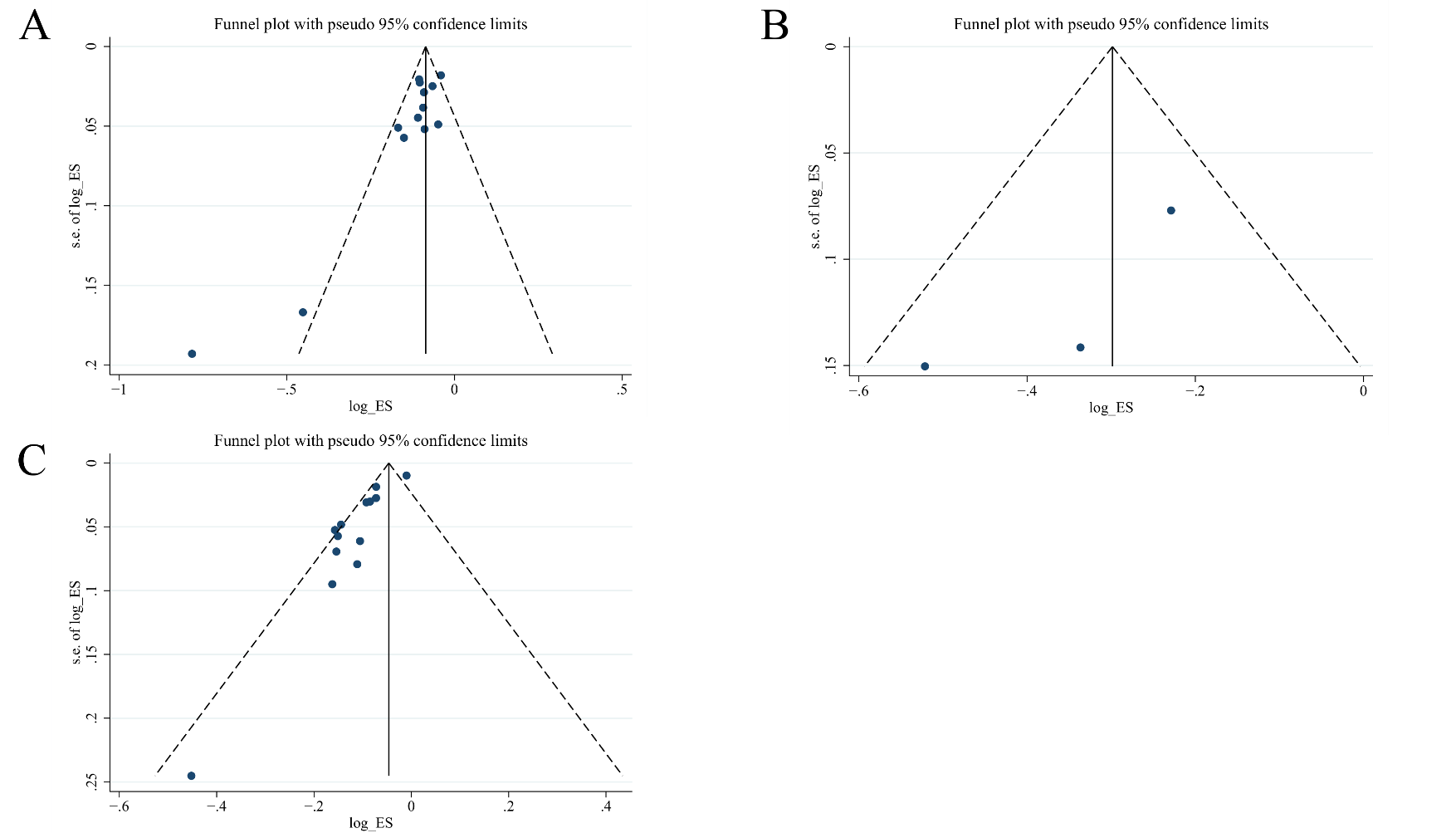


**e-Figure 16.** Funnel plot of log odd ratio (horizontal axis) and the standard error for the log odd ratio (vertical axis) for assessment of symptoms improvement after anti-reflux surgery. (A) Remission rate of heart burn. (B) Remission rate of nausea. (C)Remission rate of regurgitation pain.


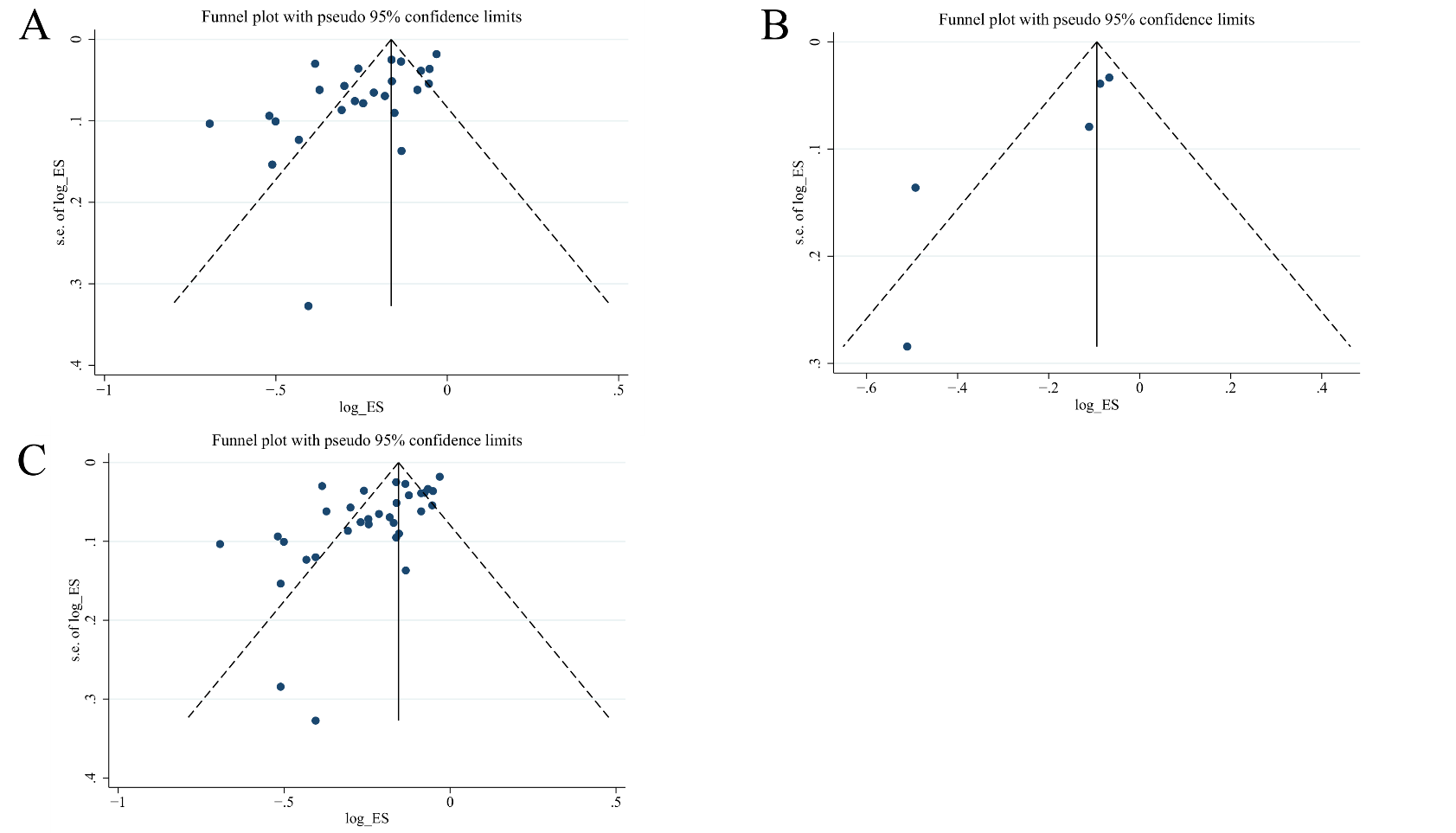


**e-Figure 17.** Funnel plot of log odd ratio (horizontal axis) and the standard error for the log odd ratio (vertical axis) for assessment of cough improvement after anti-reflux surgery. (A)Nissen. (B) Other techniques. (C) laparoscopic surgery

| **e-Table 1:** **Baseline characteristics of the study population** | |
| --- | --- |
| **Variable** | **Overall n = 7431** |
| **Male** | 2769(37%) |
| **Age** | 50.40±2.59 |
| **Follow-up (Month)** | 16.30±16.09 |
| **Patients with chronic cough** | 3577 (48%) |
| **Cough Duration (Months)** | 6.21±2.78 |
| **Patients who had surgery** | 7032 (95%) |
| **Perioperative period treatment** |  |
| **Preoperate** | 1190 |
| PPI | 916(77.0%) |
| Oral steroids | 8(0.7%) |
| Inhalative steroids and inhalative beta-2-mimetics | 14(1.2%) |
| Other | 252(21.2%) |
| **Postoperate** | 441 |
| PPI | 296(67.1%) |
| Endoscopic treatment | 25(5.7%) |
| Other | 120(27.2%) |
| **Category** | 7032(100%) |
| Laparoscopic | 2895(41%） |
| Nissen | 3985 (57%) |
| Toupet | 401 (6%) |
| Stretta | 422 (6%) |
| Hill | 277 (4%) |
| Hiatal Hernia Repair | 129 (2%) |
| Heller | 165 (2%) |
| TIF | 101 (2%) |
| Other | 233 (3%) |
| Unknown | 1293 (19%) |

**Abbreviations:** PPI, proton pump inhibitors

| **e-Table 2: Meta-regression analysis for cough remission** | |
| --- | --- |
| **Variable** | **P-value** |
| Year | 0.714 |
| No.of patients | 0.721 |
| Sex ratio | 0.894 |
